# Innate immunity mediated longevity and longevity induced by germ cell removal converge on the C-type lectin domain protein IRG-7

**DOI:** 10.1371/journal.pgen.1006577

**Published:** 2017-02-14

**Authors:** Elad Yunger, Modi Safra, Mor Levi-Ferber, Anat Haviv-Chesner, Sivan Henis-Korenblit

**Affiliations:** The Mina and Everard Goodman Faculty of Life Sciences, Bar-Ilan University, Ramat-Gan, Israel; Genentech, UNITED STATES

## Abstract

In *C*. *elegans*, removal of the germline triggers molecular events in the neighboring intestine, which sends an anti-aging signal to the rest of the animal. In this study, we identified an innate immunity related gene, named *irg-7*, as a novel mediator of longevity in germlineless animals. We consider *irg-7* to be an integral downstream component of the germline longevity pathway because its expression increases upon germ cell removal and its depletion interferes with the activation of the longevity-promoting transcription factors DAF-16 and DAF-12 in germlineless animals. Furthermore, *irg-7* activation by itself sensitizes the animals' innate immune response and extends the lifespan of animals exposed to live bacteria. This lifespan-extending pathogen resistance relies on the somatic gonad as well as on many genes previously associated with the reproductive longevity pathway. This suggests that these genes are also relevant in animals with an intact gonad, and can affect their resistance to pathogens. Altogether, this study demonstrates the tight association between germline homeostasis and the immune response of animals, and raises the possibility that the reproductive system can act as a signaling center to divert resources towards defending against putative pathogen attacks.

## Author summary

Increased sensitivity to pathogens is one of the hallmarks of aging. Thus, pathways that slow down the aging process should provide a remedy to this challenge. In this study, we have used the model organism *C*. *elegans* to identify a new longevity gene whose activation improves the resistance of the animals to pathogenic food and extends the lifespan of the animals. This improved resistance is the result of activation of the innate immune response and requires many genes known to promote longevity in animals whose germline has been removed. Nevertheless, this improved survival in the presence of pathogenic food is achieved without depleting the germline of the animals. This suggests that these longevity-promoting genes, many of which are conserved between worms and humans, can also protect animals with an intact gonad by diverting resources towards defending against putative pathogen attacks.

## Introduction

One of the most significant findings in the aging research field is the realization that lifespan is determined by the aging rate and by deleterious events that limit lifespan. Over the last few decades, a wealth of genes and signaling molecules that determine lifespan and longevity were identified. Genome-wide screens in the model organism *C*. *elegans* revealed that genes that influence aging fall into four conserved longevity pathways: the dietary restriction pathway, the insulin/IGF-1 pathway, the mitochondrial pathway and the reproductive pathway [1, 2]. In general, one common theme of these longevity regulatory pathways is their ability to shift resources towards enhanced maintenance and stress resistance of the soma [3].

In *C*. *elegans* and in *Drosophila*, germ cell removal can increase lifespan and increase resistance to oxidative stress, proteostasis stress and pathogens [4–9]. These beneficial effects of germ cell depletion require the presence of an intact somatic gonad [4, 5]. In addition, nuclear hormone signaling [5], autophagy [10–12], fat metabolism [10, 12], proteasome components [9, 13], microRNA-regulators [14] and a variety of transcription factors [5, 9–12, 15–18] are required for lifespan extension upon germ cell removal. Some of these genes (for example *daf-16*) are common mediators of several longevity pathways, whereas others are uniquely associated with the longevity of germlineless animals. Notably, many of the genes implicated in the longevity of germlineless animals act specifically in the intestine [19], which comprises the animal's adipose tissue and liver, and is also the major site of pathogen colonization in *C*. *elegans*.

Although *C*. *elegans* has evolved a dedicated innate immune response to protect the animals from infecting pathogens [20, 21], one of the factors that limit *C*. *elegans* lifespan is its slightly pathogenic microbial diet, which colonizes and infects the intestine of aging animals [22–24]. Accordingly, mutants deficient in the central innate immunity PMK-1/ATF-7 pathway exhibit a shortened lifespan even on *E*. *coli* OP50, a weakened bacterial strain that is only slightly pathogenic to *C*. *elegans* [25]. Conversely, killing or limiting the proliferation of the bacterial food source extends *C*. *elegans* lifespan [22, 26]. Furthermore, many mutations that prolong lifespan also enhance the resistance of animals to pathogenic bacteria [6, 27–30]. This enhanced resistance to pathogens is thought to be the consequence of constant expression of a variety of innate immunity-related genes [17, 30–32], which act in parallel to the PMK-1/ATF-7 innate immunity pathway [6, 30, 33]. Together, these imply that pathogen infection (even by the nonpathogenic OP50 *E*. *coli* strain) and the host immune response are important determinants of *C*. *elegans* lifespan. Here we identified the innate immunity related gene *irg-7* as a novel integral component of the reproductive longevity pathway, which links between genes implicated in this longevity pathway and pathogen resistance even in animals with an intact reproductive system.

## Results

### *irg-7(zc6)* mutants are long-lived

**Fig 1:**
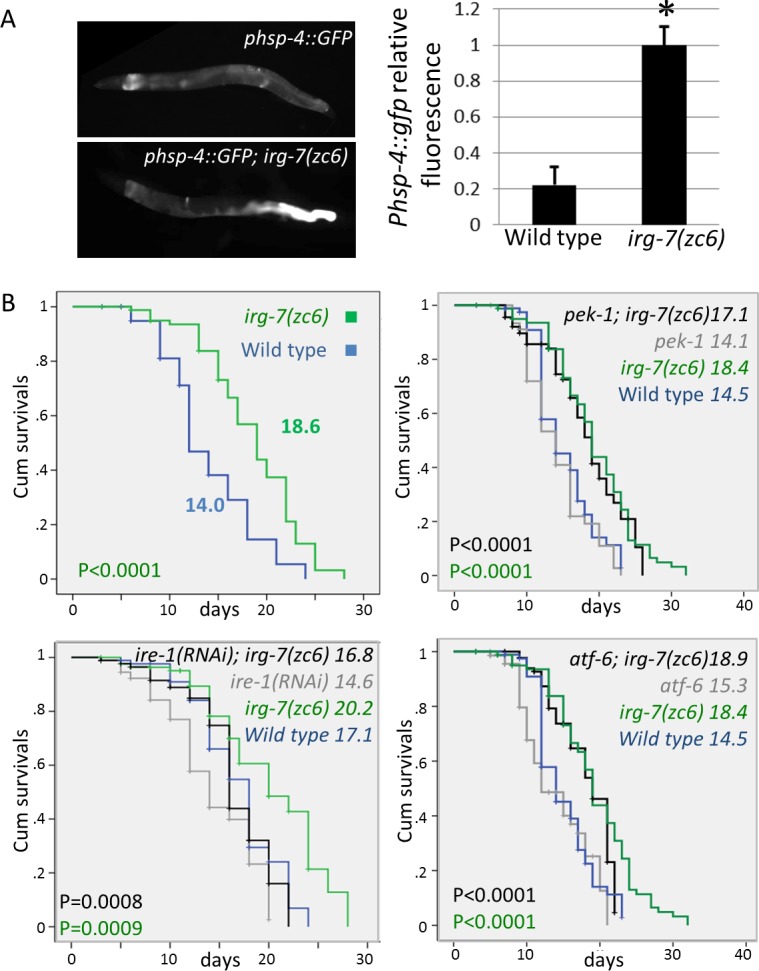
*irg-7(zc6)* mutation extends lifespan independently of UPR genes. (A) Representative fluorescence micrographs (100-fold magnification) of day-1 adults harboring an integrated *Phsp-4*::*gfp* transgene. *irg-7(zc6)* mutants expressed higher levels of the *Phsp-4*::*gfp* than did wild type animals, predominantly in the posterior end of the intestine. Asterisks mark Student's T-test values of P<0.001 compared to wild-type fluorescence. 50 animals were analyzed per genotype. Error bars represent SE of 3 independent biological replicates. (B) The *irg-7(zc6)* mutation significantly extended the lifespan of otherwise wild-type, *pek-1(ok275)*, *atf-6(ok551)* or *ire-1(RNAi)* animals. Mantel Cox P-value for the wild type vs. *irg-7(zc6)* single mutant comparison is in green. Mantel Cox P-value for the mutant vs. the mutant; *irg-7(zc6)* double mutant comparison is in black. Mean lifespan and P-values are indicated within each graph. **See S1 Table** for additional lifespan data.

One of the characteristics of the aging process is a decline in cells’ ability to mount cellular stress responses under conditions of perturbed protein homeostasis in the cytosol, in the ER and in the mitochondria [34–36]. The failure to mount these stress responses abrogates the induction of the corresponding chaperones, whose deficiency further perturbs protein homeostasis. We hypothesized that constitutive expression of chaperones may be beneficial for maintaining proteostasis in aged animals, and thus may contribute to their health-span and their longevity. To test this, we assessed the lifespan of *irg-7(zc6)* mutants, in which the *hsp-4* ER resident chaperone is constitutively expressed in the animals' intestine due to a partially mapped background mutation ([37] and **Fig 1A**). This strain, which was originally named *upr-1(zc6)*, was generated as part of a seminal study by David Ron’s group, to facilitate the identification of mutations in genes required for the induction of this ER resident chaperone [37]. We note that in the absence of information about the *zc6* mutation, it was unclear whether these animals induce ER chaperone expression due to deregulation of gene expression or whether the induction is a reflection of ER stress and activation of the unfolded protein response (UPR). Either way, lifespan analysis of the *irg-7(zc6)* mutants revealed that they lived up to 30% longer than control animals (**Fig 1B and S1 Table**). With the exception of a developmental delay of 4 hours, the physiology of *irg-7(zc6)* mutants appeared to be similar to that of wild-type animals in terms of pumping rate, fecundity and progeny profile **(S1 Fig).**

If indeed the longevity of *irg-7(zc6)* mutants stems from the constitutive expression of ER-resident chaperones, whose expression is regulated by the activation of the unfolded protein response (UPR), then interference with UPR induction should curtail their lifespan. To test this, we introduced mutations in major UPR genes into the *irg-7(zc6)* strain and followed their lifespan. First, we introduced the *irg-7(zc6)* mutation into *pek-1(ok275)* and *atf-6(ok551)* mutants (harboring deletion mutations in the worm homologs of the PERK and ATF6 genes respectively). We found that the *irg-7(zc6)* mutation extended the lifespan of *pek-1(ok275)* and *atf-6(ok551)* mutants to a similar extent as it did in animals with an intact UPR (P>0.2 in Cox Regression analysis of 3 independent experiments for each genotype) (**Fig 1B** and **S1 Table**). This implies that neither of these genes is required for the longevity of *irg-7(zc6)* mutants. Surprisingly, we could not introduce the *irg-7(zc6)* mutation into *ire-1(ok799)* and *xbp-1(tm2457)* mutants (harboring deletion mutations in the worm homologs of the IRE1 and XBP1 genes respectively). Specifically, we could not detect any homozygous *xbp-1(-/-); irg-7(zc6)* or *ire-1(-/-); irg-7(zc6)* double mutants among the viable progeny of heterozygous *xbp-1(+/-)* or *ire-1(+/-) irg-7(zc6)* mutants. This suggests that some activity of the *ire-1/xbp-1* pathway is critical for the survival of *irg-7(zc6)* mutants. To circumvent this, we examined the longevity of *irg-7(zc6)* mutants treated with *xbp-1* or *ire-1* RNAi, which reduce the levels of their target genes, rather than completely eliminating them. Treatment with *xbp-1* or *ire-1* RNAi attenuated the expression of the *Phsp4*::*gfp* reporter in *irg-7(zc6)* mutants and was compatible with the survival of the *irg-7(zc6)* strain. Nevertheless, the *irg-7(zc6)* mutation still extended the lifespan of *ire-1* or *xbp-1* RNAi-treated animals to a similar extent as it did in animals with an intact UPR (P>0.3 in Cox Regression analysis of 3 independent experiments) (**Fig 1B and S1 Table**). These findings suggest that the longevity of *irg-7(zc6)* mutants does not rely on high expression levels of ER resident chaperone.

### *zc6* is a gain of function mutation in the infection response gene *irg-7*

**Fig 2:**
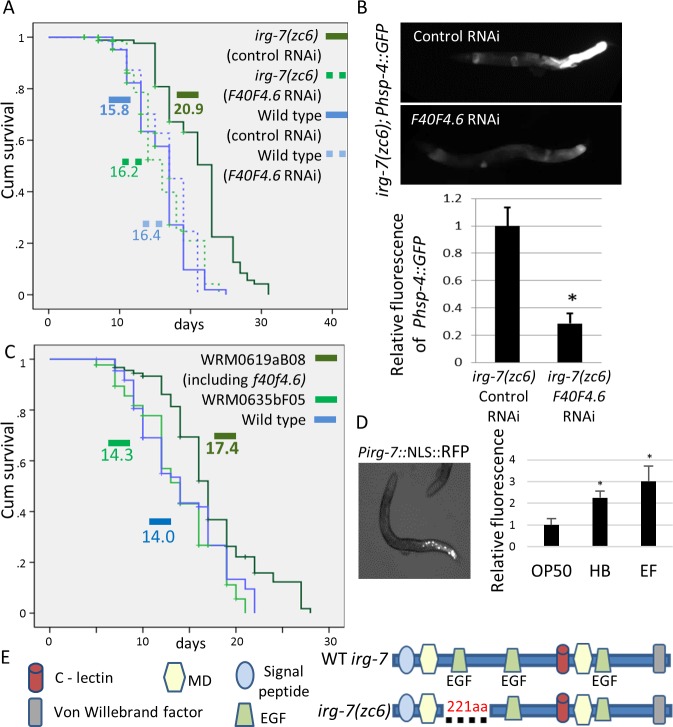
*irg-7(zc6)* is a gain of function mutation in F40F4.6. (A) *F40F4*.*6* RNAi significantly shortened the lifespan of *irg-7(zc6)* mutants (Mantel-Cox, P<0.0001) but did not affect the lifespan of otherwise wild-type animals (Mantel-Cox,P = 0.9). Mean lifespans are indicated within each graph. See **S2 Table** for more lifespan data. (B) Representative fluorescence micrographs (100-fold magnification) of day-1 adults harboring an integrated *Phsp-4*::*gfp* transgene. *F40F4*.*6* RNAi significantly reduced the expression of the *Phsp-4*::*gfp* in *zc6* mutants. Asterisks mark Student's T-test values of P<0.001 compared to fluorescence on control RNAi. 40 animals were analyzed per genotype. Error bars represent SE of 3 independent biological replicates. (C) Expression of fosmid WRM0619aB08 (which includes F40F4.6) significantly extended the lifespan of wild-type animals (Mantel-Cox, P<0.001), whereas expression of the partially overlapping fosmid WRM0635bF05 (which does not include F40F4.6) did not extend the lifespan of wild-type animals (Mantel-Cox, P = 0.4). Mean lifespans are indicated within each graph. See **S2 Table** for more lifespan data. (D) Expression of an NLS-RFP reporter fused to the putative promoter upstream of the F40F4.6 gene drives expression in the posterior cells of the intestine. Fluorescence of this reporter increased upon exposure of L4 animals to Photorhabdus luminescens subsp Hb bacteria (HB) or Enterococcus faecalis bacteria (EF). Asterisks mark Student's T-test values of P<0.001 compared to wild-type fluorescence on OP50 bacteria. (E) Schematic representation of the major domains in the F40F4.6 protein. The region deleted by the *zc6* mutation is indicated by a dashed line. This region includes an EGF domain. Note that the deletion preserves the ORF of the original gene. See S2 Fig for sequence data.

If not due to increased chaperone levels, why are *upr-1/irg-7(zc6)* mutants long-lived? An answer to this enigma may lie in the identity of the *irg-7* gene. To associate the *irg-7 zc6* mutation with its molecular and genetic identity, we completed the mapping of the *irg-7(zc6)* mutation. This mutation was previously characterized as a semi-dominant mutation located on chromosome X [37]. Using one-step whole-genome-sequencing and a SNP mapping strategy [38] we identified several candidate mutations within a mapping interval on chromosome X that may account for the *Irg-7* phenotypes. We used RNAi to knockdown these candidate genes and searched for genes whose inactivation phenocopied or suppressed the phenotypes of the *irg-7(zc6)* mutants. We found that inactivation of *f40f4*.*6* by RNAi suppressed the longevity of *irg-7(zc6)* mutants without affecting the lifespan of wild-type animals (**Fig 2A and S2 Table**). Inactivation of *f40f4*.*6* by RNAi also suppressed the expression of the ER stress reporter in *irg-7(zc6)* mutants (**Fig 2B**), suggesting that the mutation in *f40f4*.*6* is triggering the UPR in these animals. Since *f40f4*.*6* RNAi suppresses rather than phenocopies the phenotypes associated with the *irg-7(zc6)* mutation, these experiments suggest that the *irg-7(zc6)* mutation is a gain of function (gof) mutation in the *f40f4*.*6* gene.

In some cases gain of function mutations result in hyperactivation of the normal function of the encoded protein, whereas in other cases they may confer new activities. Likewise, over-expression of a protein can enhance its activity as well. Thus, we examined whether over-expression of F40F4.6 would extend lifespan similarly to the *irg-7(zc6) gof* mutation. To this end, we determined the lifespan of animals carrying a fosmid that includes the *f40f4*.*6* gene. We found that these animals lived longer than wild-type animals. In contrast, animals carrying a partially overlapping fosmid that does not include *f40f4*.*6* had a normal lifespan (**Fig 2C and S2 Table**). These experiments suggest that the *irg-7(zc6)* mutation is a gain of function mutation that enhances the normal activity of the protein.

### The *zc6* mutation removes an inhibitory domain from the infection response gene *irg-7*

Not much is known about the *irg-7* gene. Microarray studies demonstrated that the expression of *irg-7/f40f4*.*6* is induced upon exposure to some pathogens [39] as well as under dietary restriction (in this study *f40f4*.*6* has been referred to as *drd-2 [40]*). In support of these reports, we confirmed that infection by Photorhabdus luminescens subsp Hb and Enterococcus faecalis bacteria (henceforth referred to as HB and EF bacteria for brevity), induces the transcription of a reporter driven by the *irg-7* promoter in the animals intestine (**Fig 2D**).

*irg-7* encodes a protein of 2214 amino acids with a modular structure. It includes three EGF domains (that can be used for association with other regulatory proteins), a Von Willebrand factor type A domain (usually involved in adhesion via metal ion-dependent adhesion sites) and a C-type lectin domain (CTLD) (usually involved in binding to a wide variety of molecules such as sugars, proteins, lipids and inorganic compounds and implicated in pathogen recognition and clearance [41]) (**Fig 2E**). In addition, IRG-7 contains a putative signal sequence, but no putative transmembrane domain, and thus is likely to be secreted. Although each of these domains is individually evolutionarily conserved, we failed to identify any human homolog containing this exact combination of domains.

Sequencing of the *irg-7* gene in animals carrying the *zc6* mutation revealed a deletion of 901 base pairs between nucleotides 3241528–3242428 on chromosome X as well as an insertion of 2 nucleotides (**S2 Fig**). This deletion, which includes exon and intron regions, ultimately removes 221 amino acids from the protein product while preserving its open reading frame. The existence of a transcript encompassing the *zc6* mutation was confirmed by RT-PCR and sequencing (**S2 Fig**). The region included in the deletion mutation encompasses one of the EGF-like domains of the protein (**Fig 2E**). Since the *zc6* mutation is a *gof* mutation, this indicates that the deleted EGF domain is an inhibitory domain.

### *irg-7* affects pathogen resistance

**Fig 3:**
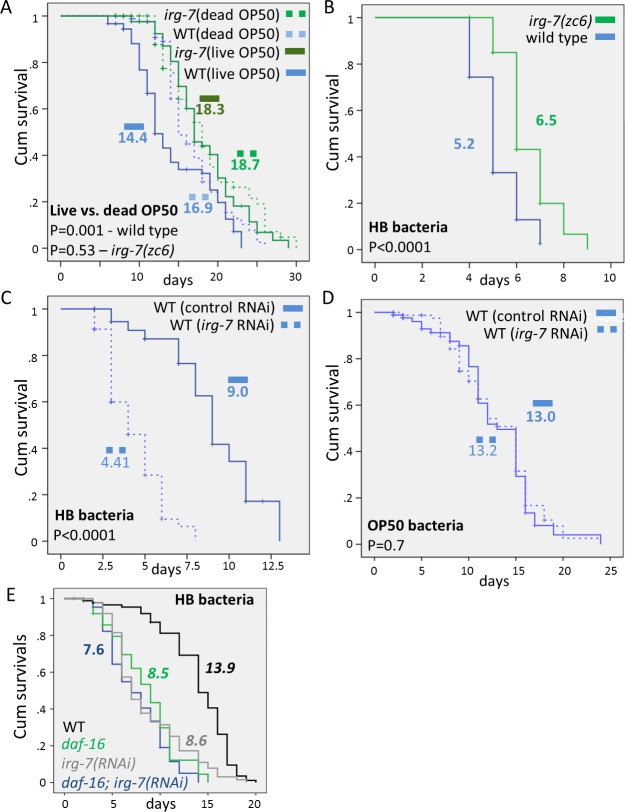
*irg-7(zc6)* mutation extends lifespan by increasing pathogen resistance. (A) Feeding animals with dead OP50 bacteria extended the lifespan of wild-type animals (Mantel-Cox, P<0.001), but did not extend the lifespan of *irg-7(zc6)* mutants (Mantel-Cox, P = 0.53). (B) The *irg-7(zc6) gof* mutation improved the survival of animals fed with pathogenic HB bacteria (Mantel-Cox, P<0.0001). (C-D) *irg-7* inactivation by pre-treatment with F40F4.6 RNAi from eggs to early adulthood hindered survival of animals fed henceforth with pathogenic HB bacteria (C) (Mantel-Cox, P<0.0001), but did not affect the survival of animals fed henceforth with OP50 bacteria (D) (Mantel-Cox, P = 0.7). (E) *irg-7* inactivation by pre-treatment with F40F4.6 RNAi from eggs to early adulthood did not affect the survival of *daf-16(mu86)* mutants fed henceforth with HB bacteria (Mantel-Cox, P = 0.43). Mean lifespan are indicated within each graph. See **S3 Table** for additional lifespan data.

One of the factors that limit *C*. *elegans* lifespan is its pathogenic food [22, 26]. CTLD-proteins have the capacity to bind carbohydrates that coat pathogens, and are induced upon contact with a variety of fungal and bacterial pathogens [41]. Thus, proteins harboring C-type lectin domains could potentially play a role in pathogen recognition and clearance [41]. Since IRG-7 harbors a CTLD domain, and since its expression is induced upon infection, we wondered whether the lifespan extension of *irg-7 gof* mutants was associated with an improved ability to deal with pathogens. Hence, we examined whether the extended lifespan of *irg-7 gof* mutants fed with live OP50 bacteria would persist if the animals were fed with killed bacteria. As previously reported [22, 26], we confirmed that the mere feeding of wild-type animals with dead bacteria extends their lifespan (**Fig 3A**). In contrast, a diet of heat-killed or UV-killed bacteria did not extend the lifespan of *irg-7 gof* mutants (**Fig 3A and S3 Table**). This implies that unlike wild-type animals, the lifespan of *irg-7 gof* mutants is not limited by the viability of its pathogenic food.

OP50 *E*. *Coli* is only slightly pathogenic to *C*. *elegans*. Thus, we next compared the survival of *irg-7 gof* mutants and wild-type animals exposed to bacteria that is more pathogenic to *C*. *elegans*. We focused on HB and EF bacteria, as these pathogenic bacteria induce *irg-7* expression ([41] and **Fig 2D**). We found that the *irg-7 gof* mutation extended the survival of the animals exposed to HB or EF bacteria (**Fig 3B and S3 Table**). Furthermore, treatment with *irg-7* RNAi during larval development significantly compromised the survival of animals exposed to HB bacteria during adulthood (**Fig 3C and S3 Table**). This implies that the *irg-7* gene normally promotes the survival of wild-type animals exposed to these pathogenic bacteria. Nevertheless, treatment with *irg-7* RNAi during larval development did not affect the survival of animals fed with OP50 during adulthood (**Fig 3D and S3 Table**). The lack of lifespan shortening by *irg-7* RNAi in animals fed with OP50 bacteria, as opposed to the curtailed survival of the *irg-7* RNAi-treated animals on the HB bacteria, supports the conclusion that the diminished survival is a result of pathogen sensitivity rather than an aging-related phenotype. These results, together with a consideration of its structural molecular domains, implicate *irg-7* in pathogen resistance.

### *irg-7 gof* activates the immune response

**Fig 4:**
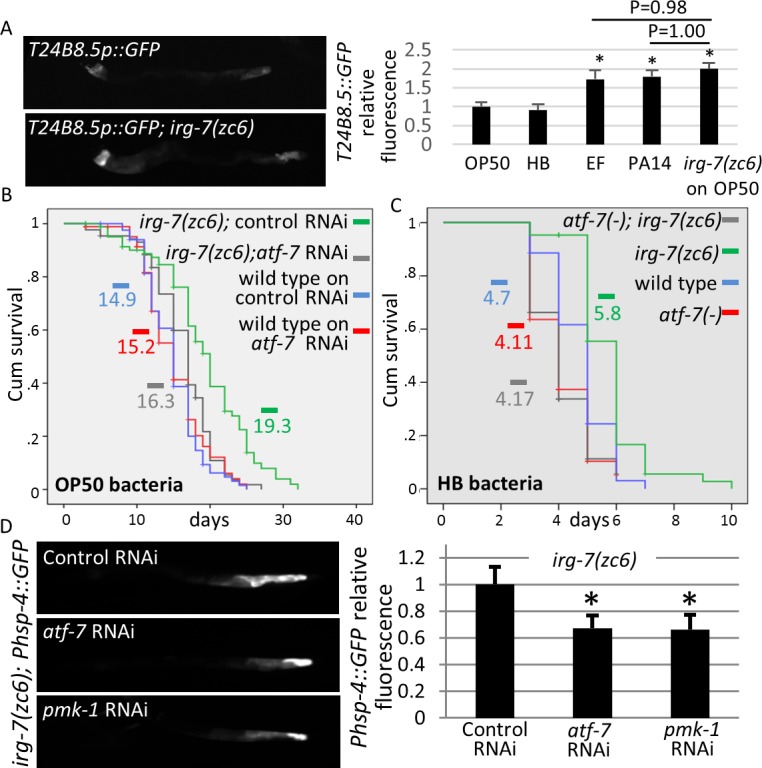
The innate immunity-related transcription factor ATF-7 promotes longevity and pathogen resistance in *irg-7(zc6)* mutants. (A) The *irg-7(zc6)* mutation increases the expression of the *T24B8*.*5*::*gfp atf-7* target gene reporter (Mann-Whitney, P<0.0001). This increased expression by *irg-7(zc6)* is similar to that induced by exposure to HB or EF bacteria (Mann-Whitney, P = 1.00 and P = 0.98 respectively). Asterisks mark Mann-Whitney values <0.001 compared to the fluorescence in wild-type animals on OP50 bacteria. 30 animals were analyzed per genotype. Error bars represent SE of 3–5 independent biological replicates. (B) The *irg-7(zc6)* mutation did not extend the lifespan of animals treated with *atf-7* RNAi (Mantel-Cox, P = 0.07). Mean survival is indicated within the graph. See **S4 Table** for additional lifespan data. (C) *atf-7(gk715); irg-7(zc6)* double mutants animals did not survive on HB bacteria better than *atf-7(gk715)* single mutants (Mantel-Cox, P = 0.9). Mean survival is indicated within the graph. See **S4 Table** for additional survival data. (D) Representative fluorescence micrographs (100-fold magnification) of day-1 adults harboring an integrated *Phsp-4*::*gfp* transgene. *atf-7* and *pmk-1* RNAi significantly reduced the expression of the *Phsp-4*::*gfp* in *irg-7(zc6)* mutants. Asterisks mark Student's T-test values of P<0.002 compared to fluorescence on control RNAi. 30 animals were analyzed per genotype. Error bars represent SE of 3 independent biological replicates.

In principle, the IRG-7 protein could enhance pathogen resistance by directly neutralizing pathogenic bacteria (for example via its specialized CTLD domain which can directly bind to sugars encoating bacteria and perforate their cell membrane [42] or cause their spatial segregation of microbiota and the host intestine [43]). Alternatively, it may do so indirectly, by activating the animals’ immune response. One of the central signaling cascades of the innate immune response in *C*. *elegans* is the PMK-1/ATF-7 pathway [20, 21]. Thus, we examined whether the *irg-7 gof* mutation recruited this innate immunity pathway to the defense and well-being of the organism. To this end, we followed the expression of a GFP reporter driven by the promoter of *T24B8*.*5*, an ATF-7-target gene [20]. We found that the levels of the ATF-7 reporter increased in *irg-7 gof* mutants compared to animals with wild-type *irg-7* (Mann-Whitney P<0.0001, **Fig 4A**). The level of the ATF-7 reporter in *irg-7 gof* mutants were the same as in animals exposed to EF (Mann-Whitney, P = 0.98) and PA14 pathogens (Mann-Whitney, P = 1.00) (**Fig 4A**). Interestingly, exposure to HB bacteria did not increase the expression of this ATF-7 reporter (Mann-Whitney, P = 1.00) (**Fig 4A**). These findings are consistent with the interpretation that the PMK-1/ATF-7 innate immunity pathway is activated in *irg-7 gof* mutants.

Next, we examined whether the increased expression of the ATF-7 reporter simply correlated with the *irg-7(zc6)* mutation or whether it is important for the longevity of *irg-7 gof* mutants. In support of the latter, we found that the longevity of *irg-7 gof* mutants on live OP50 was completely dependent upon the integrity of the innate immunity PMK-1/ATF-7 pathway (Mantel-Cox P>0.05 in six independent experiments—**Fig 4B, and S4 Table**). Similarly, the improved survival of *irg-7 gof* mutants on the pathogenic HB bacteria was also dependent upon the PMK-1/ATF-7 innate immunity pathway (Mantel-Cox P>0.05 in three independent experiments) (**Fig 4C and S4 Table**). Altogether, these findings support the conclusion that *irg-7* activation acts as an immune-modulator, which engages the nematode's innate immune response and improves the ability of animals to cope with the pathogenic food.

### The ER stress response is constitutively active in *irg-7 gof* mutants because of their immune response

One consequence of activation of the innate immune response is an increased load on the ER, presumably due to massive production of secreted antibacterial proteins [44]. Since the ER stress response is constitutively activated in the intestine of *irg-7 gof* mutants (**Fig 1A**, [37]), we wondered whether this stress response may be due to the activity of the PMK-1/ATF-7 innate immunity pathway in these mutants. Consistent with this possibility, inactivation of *pmk-1* or *atf-7* reduced the levels of the *Phsp-4*::*gfp* ER-stress reporter in the intestine of the *irg-7 gof* animals (Student’s T-test P<0.002, **Fig 4D**). Thus, the PMK-1/ATF-7 innate immunity pathway appears to contribute to the induction of the UPR in *irg-7 gof* mutants.

### *irg-7* is required for lifespan extension upon germline removal

**Fig 5:**
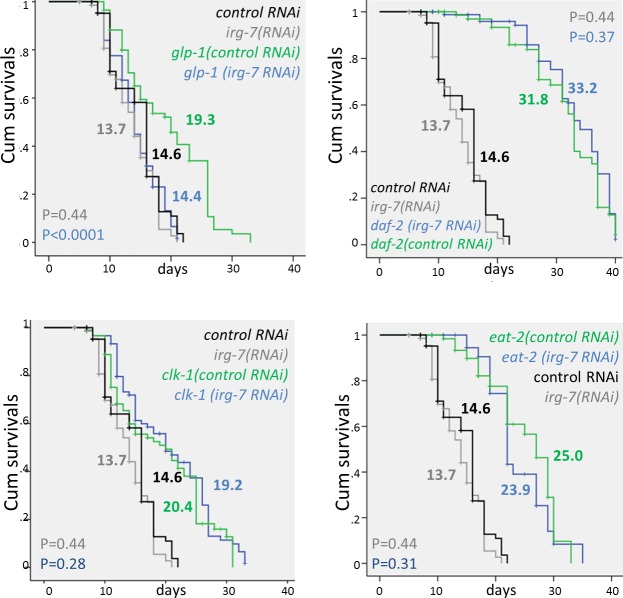
*irg-7* is part of the germline longevity pathway. *F40F4*.*6* RNAi significantly shortened the lifespan of *glp-1* germlineless animals, but did not shorten the long lifespan of insulin/IGF1 signaling mutants (*daf-2*), dietary restricted mutants (*eat-2)*, mutants in mitochondrial respiration mutants (*clk-1*) or wild-type animals. Mean lifespans and Mantel-Cox P values are indicated within each graph. Mantel Cox P-values for control RNAi vs. *irg-7* RNAi-treated wild-type animals are in gray. Mantel Cox P-values for control RNAi vs. *irg-7* RNAi-treated long-lived mutants are in blue. See **S5 Table** for more lifespan data.

Many longevity pathways confer increased pathogen resistance [6, 27–30]. Hence we wondered whether the *irg-7* gene is an integral part of any of the known longevity pathways. To this end, we compared the lifespan of wild-type animals and various long-lived mutants upon treatment with control or *irg-7* RNAi. Using this approach, we found that *irg-7* RNAi suppressed the extended lifespan of *glp-1* mutants which lack a germline (Mantel-Cox P<0.0001 in 3 independent experiments, **Fig 5 and S5 Table**), although it had no effect on the lifespan of wild-type animals (Mantel-Cox P>0.2 in 4 independent experiments, **Fig 5 and S5 Table**). *irg-7* RNAi did not affect the lifespan of animals that are long-lived due to reduced insulin/IGF1 signaling (i.e. *daf-2* mutants), due to reduced mitochondrial respiration (*clk-1* mutants) or due to a mutation in the *eat-2* gene (a mutation in an acetyl-choline receptor that causes reduced pharyngeal pumping and extended longevity) (Mantel-Cox P>0.2 in 3 independent experiments for each long-lived strain, **Fig 5 and S5 Table**). The observation that *irg-7* inactivation had only mild effects on wild-type lifespan, but almost completely prevented germline loss from extending lifespan, suggests that *irg-7* plays a regulatory role specifically in this pathway.

### *irg-7* transcription is increased upon germ cell removal

**Fig 6:**
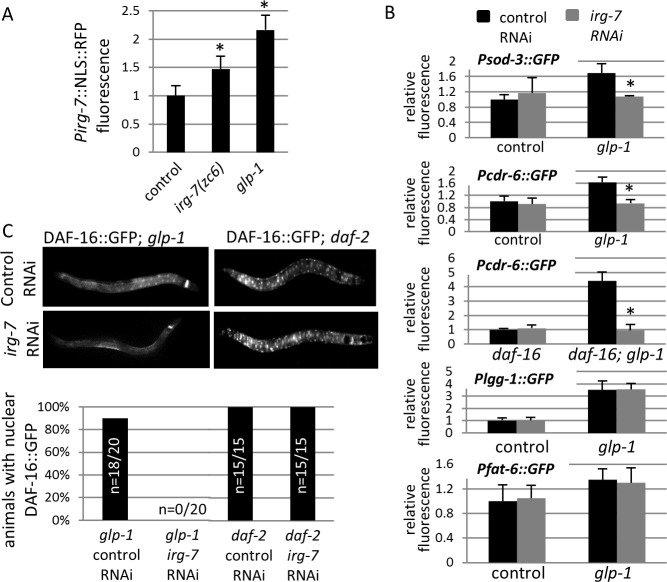
*irg-7* is required for DAF-12 and DAF-16 activation in germline-less animals. (A) Expression of an NLS-RFP reporter fused to the promoter of upstream of the *F40F4*.*6* gene is induced in *irg-7(zc6)* mutants and upon germ cell removal (*glp-1*). Asterisks mark Student's T-test values of P<0.005 compared to the fluorescence in wild-type animals. 30 animals were analyzed per genotype. Error bars represent SE of 3 independent biological replicates. (B) F40F4.6 RNAi reduced the expression of the DAF-16 and DAF-12 reporters *Psod-3*::*gfp* and *Pcdr-6*::*gfp* in germline-less animals, but did not affect the expression of the PHA-4 and NHR-80 reporters *Plgg-1*::*gfp* and *Pfat-6*::*gfp*. Asterisks mark Mann-Whitney P values <0.0001 compared to the fluorescence in control RNAi-treated animals. 30 animals were analyzed per genotype. Error bars represent SE of 3 independent biological replicates. (C) *F40F4*.*6* RNAi reduced DAF-16::GFP nuclear translocation in germline-less animals (left panels) but not in *daf-2(e1370)* mutants (right panels).

After implicating *irg-7* in the germline longevity pathway, we wondered whether its expression is regulated by this pathway. To this end, we examined whether the expression levels or expression pattern of *irg-7* changes upon germ cell removal. Specifically, we compared the expression of the *irg-7* transcription reporter in animals with a normal reproductive system and in animals with no germ cells. In both cases, the reporter driven by the *irg-7* promoter was expressed specifically in the posterior intestinal cells, regardless of the presence or absence of the germ cells. However, the fluorescence levels of the transcriptional reporter increased upon germ cell removal (Student’s T-test P<0.005, **Fig 6A**). This indicates that *irg-7* is associated with the germline longevity pathway in at least two ways. First, it is required for the longevity of germlineless animals. Second, its' transcription is regulated by the reproductive tissues. Interestingly, a similar increase in the levels of the transcriptional reporter was also observed in *irg-7(zc6)* mutants, suggesting that *irg-7* activation indirectly promotes its own transcription as part of an auto-regulatory loop (Student’s T-test P<0.005, **Fig 6A**).

### *irg-7* is required for activation of parts of the transcriptional program in germlineless animals

Since the germline longevity pathway engages many transcription factors, and *irg-7* is a new factor in the germline longevity pathway, we examined whether the transcriptional network that promotes longevity in germlineless animals is affected by *irg-7* depletion. To this end, we compared the induction of a set of reporters known to be induced by different transcription factors upon germ cell removal. We found that the *fat-6* and *lgg-1* reporters, target genes of the NHR-80 and PHA-4 transcription factors respectively [10, 15], were induced upon germ cell removal regardless of treatment with *irg-7* RNAi (**Fig 6B**). In contrast, *irg-7* inactivation compromised the induction of the reporters of *sod-3* (a target gene of DAF-16) [45] and *cdr-6* (a target gene of DAF-16 and DAF-12 [46, 47]) upon germ cell removal (**Fig 6B**).

Since *irg-7* inactivation interfered with the induction of two *daf-16* target genes in germlineless animals we hypothesized that *irg-7* might be required for the activation of this transcription factor upon germ cell removal. A critical step in the activation of DAF-16 upon germ cell removal is its nuclear translocation in the intestine cells [45]. Thus, we used animals expressing fluorescently tagged DAF-16 to explore whether *irg-7* was required for this step. We could not detect translocation of DAF-16::GFP to the nuclei of the intestine cells upon germ cell removal in animals treated with *irg-7* RNAi, (**Fig 6C**).

The translocation of DAF-16 from the cytoplasm to the nucleus can be triggered by a variety of signals [48]. Hence, we wondered whether *irg-7* inactivation globally abrogates DAF-16’s ability to translocate from the cytoplasm to the nucleus, or is it only impaired in response to a subset of signals (as in the case of germline depletion). To this end, we followed the affect of *irg-7* RNAi on DAF-16’s intra-cellular localization in animals with reduced insulin/IGF1 signaling. We confirmed that when insulin signaling is perturbed, DAF-16::GFP translocates to the nuclei of all cells. However, in contrast to its interference with DAF-16::GFP’s translocation to the nuclei in germlineless animals, *irg-7* RNAi did not affect the accumulation of DAF-16::GFP in the nuclei of insulin/IGF-1 signaling mutants (**Fig 6C**). This indicates that *irg-7* is specifically required for the nuclear translocation of DAF-16 upon germ cell removal.

Inactivation of *irg-7* also suppressed the induction of the *cdr-6* reporter (**Fig 6B**). The transcription of the *cdr-6* reporter is independently and additively induced upon germ cell removal by either the DAF-16 or the DAF-12 transcription factors [47]. In order to uncouple between the two, we examined the expression of the *cdr-6* reporter in *daf-16*-deficient animals, which can still induce *cdr-6* expression upon germ cell removal in a *daf-12*-dependent manner. We found that *irg-7* RNAi suppressed the induction of the *cdr-6* reporter upon germ cell removal in this background as well (**Fig 6B**). This indicates that *irg-7* is an important regulator of the germline longevity pathway, which alters the transcriptional outputs of the DAF-16 and DAF-12 transcription factors upon germ cell removal.

### The longevity of *irg-7* gof mutants and longevity induced by germ cell removal depend on many common factors

**Fig 7:**
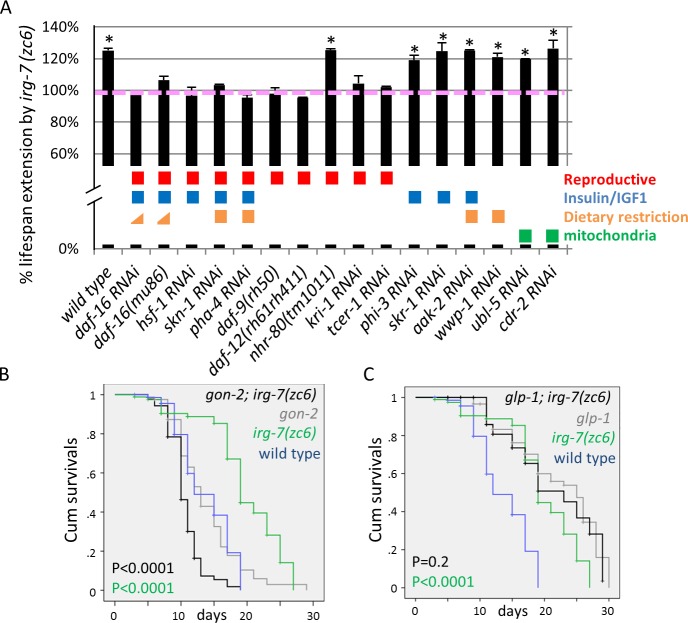
The longevitiy induced by germ cell removal and by the *irg-7(zc6)* mutation extend lifespan by a shared mechanism. (A) Bar graph presenting the percentage of lifespan extension conferred by the *irg-7(zc6)* mutation in the indicated genetic backgrounds. Each bar is the average of at least three independent lifespans. Error bars represent SE of at least 3 independent biological replicates. Asterisks mark bars in which all the lifespan experiments were increased significantly by the *irg-7(zc6)* mutation (P<0.05). Colored boxes indicate longevity pathways associated with each longevity gene: the reproductive longevity pathway induced by germ cell removal (Red), the insulin/IGF1 signaling pathway (Blue), the dietary restriction pathway (Orange) and the mitochondrial respiration pathway (Green). Note that the dietary restriction pathway is only partly dependent of *daf-*16 (marked with an Orange triangle), depending on the dietary regimen used. Note that the longevity of *irg-7(zc6) gof* mutants requires many genes implicated in the longevity of germline-less animals with the exception of *nhr-80*, which is not required for *irg-7(zc6)* longevity although it is required for the longevity of germlineless animals. See **S6 Table** for more lifespan data. (B-C) The *irg-7(zc6) gof* mutation extended the lifespan of wild-type animals with an intact reproductive system but shortened the lifespan of animals that lack a somatic gonad (B). It did not further extend the lifespan of germline-less animals (C). Mean lifespan and Mantel-Cox P-values are indicated within each graph. Mantel Cox P-value for the wild type vs. *irg-7(zc6)* single mutant comparison is in green; Mantel Cox P-value for the mutant vs. the mutant; *irg-7(zc6)* double mutant comparison is in black. **See S6 Table** for additional lifespan data.

After establishing that *irg-7 gof* mutants are long-lived, we explored which known lifespan-related genes are required for their longevity. Specifically, we used RNAi or deletion mutants to inactivate the expression of known longevity genes and then examined the ability of the *irg-7* mutants to live long. We found that the increased lifespan of *irg-7 gof* mutants required most of the genes previously associated with the reproductive longevity pathway (**Fig 7A and S6 Table**). Some of these genes are uniquely associated with the reproductive longevity pathway (*tcer-1 [49], kri-1 [50], daf-9, daf-12* [5]). Others are common to the reproductive longevity pathway and other longevity pathways (e.g. *skn-1* [17, 51, 52], *daf-16 [5, 45, 53, 54], hsf-1[2, 55], aak-2 [56]* and *pha-4 [10, 57]* which are also a part of the dietary restriction and/or insulin/IGF1 signaling longevity pathways). Silencing of additional genes, required for insulin/IGF-1-induced longevity but not required for the longevity of germline-less animals (*phi-3*, *skr-1* [13]), did not shorten the longevity of *irg-7* mutants by more than 5% (**Fig 7A and S6 Table).** Likewise, silencing of genes required uniquely for the mitochondrial longevity pathway (*cdr-2*, *ubl-5* [58, 59]), and/or the caloric-restriction longevity pathway (*wwp-1* [60]), did not shorten the longevity of *irg-7 gof* mutants by more than 5% (**Fig 7A and S6 Table**).Thus, *irg-7*-mediated longevity appears to be related to the longevity mediated by germ cell removal, as the two rely on many common genes.

Lifespan extension by germ cell removal requires the presence of the somatic gonad [5, 47]. Thus, we examined whether lifespan extension by the *irg-7 gof* mutation requires the somatic gonad as well. We found that instead of extending lifespan, the *irg-7 gof* mutation shortened lifespan in the absence of a somatic gonad (**Fig 7B and S6 Table**). Thus, *irg-7* activation, similarly to germ cell removal, relies on the somatic gonad to promote longevity.

Finally, we examined the effect of the *irg-7(gof)* mutation on the longevity of animals that lack germ cells but have a somatic gonad. We found that the *irg-7 gof* mutation did not increase the lifespan of germlineless animals (**Fig 7C and S6 Table**). This further supports the notion that the *irg-7* activation and germ cell removal increase lifespan by a common mechanism, which relies on many common genes and on the somatic gonad.

### *irg-7 gof* partially phenocopies the transcriptional reprogramming associated with germ cell removal

Since many transcription factors are required for the longevity of germlineless animals as well as for the longevity of *irg-7(zc6) gof* mutants (**Fig 7A**), we asked whether the *irg-7 gof* mutation activates these transcription factors. Specifically, we focused on the DAF-16 and DAF-12 transcription factors, as *irg-7* was required for their activity in germlineless animals (**Fig 6B and 6C**).

**Fig 8:**
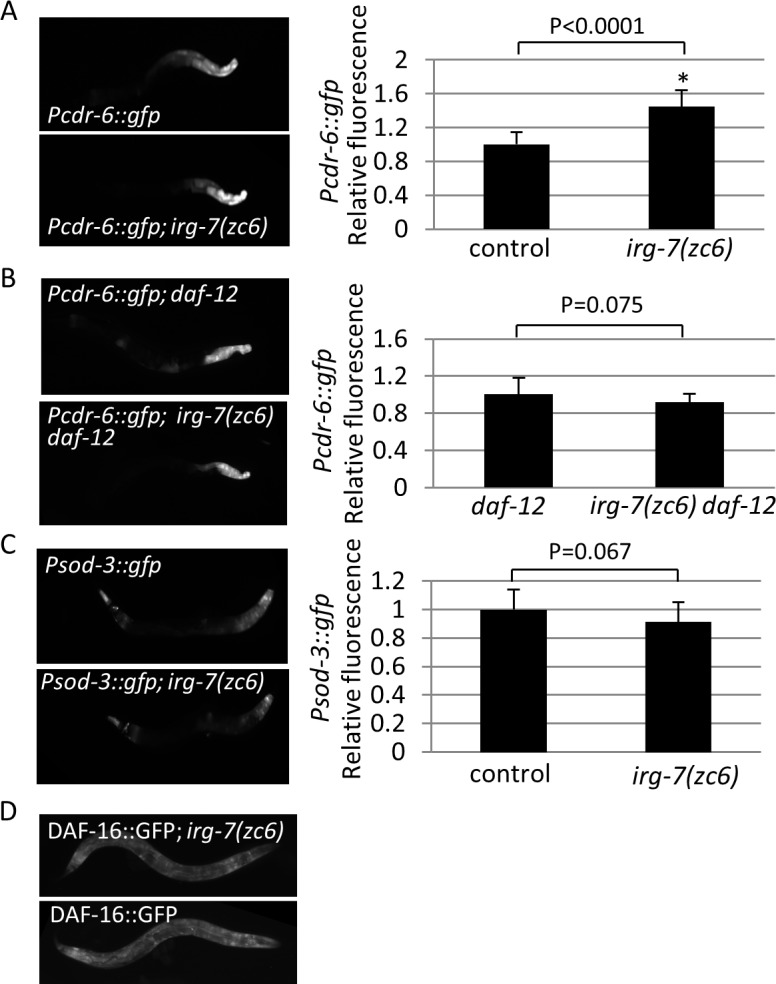
The transcription factor DAF-12 is activated in *irg-7(zc6)* mutants. (A-B) The *irg-7(zc6)* mutation is sufficient to increase the expression of the *Pcdr-6*::*gfp* reporter (Student's t-test, p<0.0001) in a *daf-12(+)* background but not in a *daf-12(-)* background (Student's t-test, p = 0.075). Asterisks mark Student's t-test values of P<0.001 compared to the fluorescence in wild-type animals. 30–35 animals analyzed per genotype. Error bars represent SE of 3 independent biological replicates. (C) The *irg-7(zc6)* mutation is not sufficient to increase the expression of the DAF-16 reporter *Psod-3*::*gfp* (Student's t-test, P = 0.067). (D) The *irg-7(zc6)* mutation is not sufficient for the accumulation of DAF-16::GFP translational reporter in the intestine.

First, we followed the levels of a *cdr-6* transcriptional reporter, which is transcribed both by DAF-16 and by DAF-12. We found that the *irg-7 gof* mutation increased the level of the reporter in animals with an intact reproductive system by 1.5 fold (Student’s T-test P<0.0001, **Fig 8A**). This increase was dependent on the *daf-12* transcription factor, as it was not observed in its absence (Student’s T-test P = 0.075, **Fig 8B**). Thus, *irg-7 gof* activates the longevity-associated transcription factor DAF-12.

Next, we followed the levels of a *sod-3* transcriptional reporter, which is a direct target gene of the DAF-16 transcription factor. Surprisingly, we did not detect an increase in the level of the *sod-3* reporter in *irg-7 gof* mutants (Student’s T-test P = 0.067, **Fig 8C**). Likewise, we did not observe accumulation of DAF-16::GFP in the nuclei *of irg-7(zc6)* animals (**Fig 8D**). This could indicate that *irg-7 gof* is not sufficient to promote the nuclear translocation of the DAF-16 transcription factor to the nucleus, nor is it sufficient to significantly increase DAF-16 activity. Nevertheless, DAF-16 was required for the lifespan extension in *irg-7 gof* mutants (**Fig 7A**). Furthermore, DAF-16 was required for the improved survival of *irg-7 gof* mutants exposed to pathogenic HB bacteria (**Fig 3E and S3 Table**). These suggest that the basal activity of DAF-16, which is below the sensitivity of this assay, but is not completely absent, is critical for the beneficial phenotypes of *irg-7(zc6) gof* mutants. Alternatively, it could be that the transcriptional targets of DAF-16 in animals with activated *irg-7* are distinct from those examined here.

### Germline homeostasis is modulated by HB bacteria and contributes to pathogen resistance

**Fig 9:**
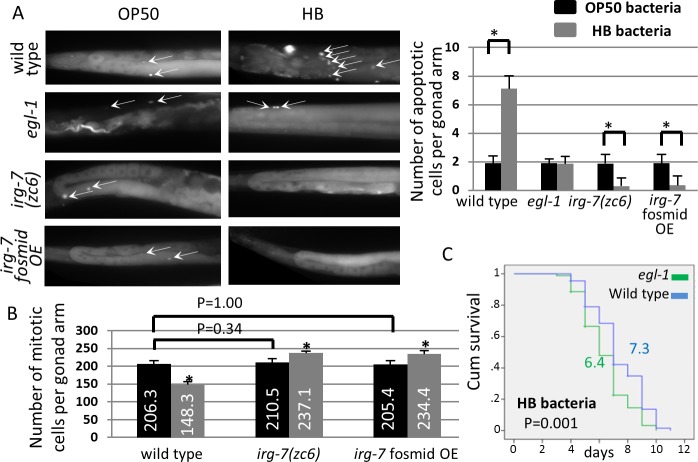
Sensitivity of germline homeostasis to pathogenic HB bacteria improves the animals' survival. (A) Treatment with pathogenic HB bacteria induced germline apoptosis in an *egl-1* dependent manner. Asterisks mark Mann-Whitney P values of P<0.001 of HB-treated animals (gray) compared to OP50 treated animals (black) of the same genotype. 45–50 animals were analyzed per genotype. Error bars represent SD. (B) Amount of mitotic germ cells was determined in wild-type animals and in animals with activated *irg-7* upon exposure to OP50 (black) or HB bacteria (gray). Asterisks mark Mann-Whitney values of P<0.001 of HB-treated animals (gray) compared to OP50 treated animals (black) of the same genotype. Additional Mann-Whitney P values are indicated in the graph. Note that treatment with pathogenic HB bacteria reduced the amount of mitotic germ cells in the gonad of wild-type animals but not in animals with activated *irg-7*. Also note that activation of *irg-7* did not reduce, and even increased, the amount of mitotic germ cells in the animals. 25–30 DAPI-stained gonads were analyzed per genotype. Error bars represent SE of 3 independent biological replicates. (C) In the absence of pathogen-induced germline apoptosis, animals are more sensitive to the pathogenic bacteria. Mean survival and Mantel-Cox P-values are indicated within the graph. See **S7 Table** for additional survival data.

We found that *irg-7* is implicated in pathogen resistance on one hand, and is an integral component of the reproductive longevity pathway on the other hand. Hence, we wondered whether these two processes are linked. Therefore, we asked whether germline physiology is modulated in the presence of pathogens. To this end, we examined whether germline homeostasis is modulated by the pathogenic HB bacteria. We found that exposure to HB bacteria increased germ cell apoptosis (Mann-Whitney P<0.0001, **Fig 9A**) and decreased the number of mitotic germ cells (Mann-Whitney P<0.0001, **Fig 9B**) in the gonads of wild-type animals. To see if these changes in germline homeostasis contributed to the resistance of the animals to the pathogen, we decided to block some of these changes and to see if this increases the sensitivity of the animals to the pathogen. To this end, we used *egl-1* mutants, which block germline apoptosis induced by *Salmonella typhimurium* [61]. As in the case of *Salmonella typhimurium*-induced germline apoptosis [61], a mutation in the *egl-1* gene blocked HB bacteria-induced germline apoptosis (Mann-Whitney P = 1.00, **Fig 9A**) and increased the sensitivity of the animals to pathogen exposure (Mantel-Cox P<0.02 in 3 independent experiments, **Fig 9C and S7 Table**). These findings raise the possibility that the germline may serve as a tissue-level pathogen sensor whose depletion can promote animals’ survival in the presence of pathogens.

### *irg-7* activation does not affect germline homeostasis when animals are fed with OP50 bacteria

Exposure to pathogenic bacteria increases the expression of the *irg-7* gene and alters germline homeostasis. Hence, we examined whether these events occur consecutively such that pathogen exposure leads to activation of *irg-7*, which in turn affects germline homeostasis. To this end, we examined whether activation of *irg-7* is sufficient to affect germline homeostasis. *irg-7* activation was achieved by introducing the *irg-7 gof* mutation or by over-expressing multiple copies of a fosmid that includes the wild-type *irg-7* gene in animals fed with OP50 bacteria. We found that the levels of germ cell apoptosis and the amount of mitotic germline in these animals were similar to those of wild-type animals (Mann-Whitney P>0.3, **Fig 9A and 9B**). This indicates that under these conditions, germline homeostasis is not regulated by *irg-7* activation. This once again indicates that *irg-7* does not mediate its beneficial effects on OP50-fed animals by acting upstream to the germline. Rather, since *irg-7* expression is induced upon germ cell removal, it should be placed downstream to the perturbations in germline homeostasis.

### *irg-7* activation protects germline homeostasis upon exposure to HB bacteria

In addition to the beneficial lifespan affects conferred by *irg-7* activation in animals fed with OP50 bacteria, *irg-7* activation also improves the survival of animals on pathogenic bacteria. Hence, we decided to examine whether under these conditions (upon exposure to pathogenic bacteria) *irg-7* activation leads to perturbations in germline homeostasis. We found that that even in the presence of HB bacteria, no increase in germline apoptosis or reduction in the amount of mitotic germ cells was observed in animals with activated *irg-7*. Unexpectedly, the mitotic germline of animals with activated *irg-7* exposed to HB bacteria was even more expanded than prior to their exposure to pathogenic bacteria (Mann-Whitney P<0.0001, **Fig 9B)**. Likewise, barely any apoptotic germ cells were detected in the gonads of animals with activated *irg-7* upon treatment with HB bacteria (**Fig 9A**). Whereas the reasons for the robustness of germline homeostasis in *irg-7* mutants is not known, an appealing speculation is that *irg-7* activation successfully recruited the innate immune response to protect the animals from the pathogenic food and its toxic interaction with the animals’ germline.

## Discussion

Previous studies have demonstrated that signals from reproductive tissues influence longevity, yet only a fraction of the underlying genetic network that controls this process has been elucidated. In this study, we identified *irg-7*, as an integral downstream component of the germline longevity pathway.

*irg-7* encodes an intestinally-produced secreted protein, harboring a single CTLD domain (typically associated with proteins involved in innate immunity anti-microbial activity) and several EGF domains. Interestingly, removal of one of these EGF domains activates the protein, assigning it as an auto-inhibitory domain. This auto-inhibition of IRG-7 is reminiscent of toxic mammalian CTLD-harboring proteins, which contain intramolecular inhibitory domains that maintain them in an inert inactive state, which can be switched into an active state by proteolytic removal of the inhibitory segment [42, 62, 63].

Our initial interest in *irg-7(zc6)* mutants was because of the constitutively high expression of the *hsp-4* ER resident chaperone in their intestine. At first, it was unclear whether these animals induce ER chaperone expression due to deregulation of gene expression or as a reflection of ER stress and activation of the UPR. Our findings indicate that the hyperactivation of the ER stress response in these animals is a reflection and a consequence of their activated innate immune response. The UPR-mediated coordination between the secretory capacity and secretory load is critical in animals with an activated immune response. In the absence of such coordination, activation of the innate immune response is detrimental [44]. Consistent with this, complete depletion of the UPR genes *ire-1* and/or *xbp-1* using deletion mutants was lethal for *irg-7* gof mutants, whose innate immune response is activated. Nevertheless, *irg-7 gof* mutants survived *ire-1* and/or *xbp-1* RNAi treatment. Furthermore, these RNAi treatments did not compromise their longevity. This dichotomy may indicate that a low level of UPR activity is sufficient to provide the basic adaptation required by the over-loaded ER in animals with an activated innate immune response. This residual UPR activity may reflect low levels of UPR activity throughout the animal. Alternatively, it may reflect UPR activity in specific tissues, most likely in neurons, which are relatively resistant to RNAi treatment. This mechanism is consistent with previous findings that neuronal signaling can modulate the innate immune response in the *C*. *elegans* intestine [64, 65].

We consider *irg-7* as an integral downstream component of the germline longevity pathway because its expression is increased upon germ cell depletion and because it is essential for the longevity of germlineless animals. In germlineless animals, depletion of *irg-7* interferes with the activation of the longevity-promoting transcription factors DAF-16 and DAF-12. At the same time, *irg-7* deficiency does not affect germ cell regulated activity of the transcription factors NHR-80 and PHA-4. Thus, at least two independent signaling pathways control the transcriptional network that is set upon germ cell depletion; only one of which implicates *irg-7*, which acts upstream or in parallel to *daf-16* and *daf-12*. These perturbations are enough to preclude lifespan extension by germ cell depletion.

Although inactivation of *irg-7* does not shorten the lifespan of animals with an intact reproductive system, its’ activation extends the lifespan of the animals. The lifespan extension induced by *irg-7* activation requires the presence of the somatic gonad, similarly to the longevity conferred by germ cell removal. In addition, it shares many genetic determinants with the reproductive longevity pathway. Although there is an extensive overlap between genes required for the lifespan extension induced by *irg-7* activation and those required for longevity induced by germ cell depletion, the lifespan extension induced by *irg-7* activation does not perturb germline homeostasis under normal growth conditions. Thus, the lifespan extension by *irg-7* activation is independent of germline depletion, yet requires many of the same longevity genes as in germlineless animals.

How does *irg-7* activation extend lifespan? Many CTLD proteins, which are abundant in the nematode genome (~280 genes), show a pathogen-specific induction during infection and are thought to be important for enhancing the nematode's ability to fight off pathogens [66, 67]. Furthermore, the expression of several CTLD proteins is regulated by innate immunity pathways [6, 33, 67, 68]. Our data implicates *irg-7* in regulating the animals’ sensitivity to pathogens in several ways. First, although the lifespan of wild-type animals is limited by its pathogenic food, in *irg-7(zc6)* mutants, lifespan was unaltered whether the animals were fed with live or with dead OP50 bacteria. Second, *irg-7* activation promotes the survival of animals on pathogenic HB bacteria, whereas its inactivation renders wild-type animals more sensitive to the same pathogen. Third, *irg-7* activates and engages the *pmk-1/atf-7* innate immune response pathway to the defense and well-being of the animals and requires this innate immunity pathway for lifespan extension. Altogether, these imply that *irg-7*-associated life extension is tightly associated with its enhanced ability to defend the animals from the pathogenic toxicity of its food.

Since *irg-7* is induced in germlineless animals, and since its induction can lead to activation of the PMK-1/ATF-7 innate immunity pathway, our findings suggest that the canonical PMK-1/ATF-7 pathway may also be activated in germlineless animals. Consistent with this, an increase of 1.3 fold in the transcript levels of several *atf-7* target genes has been observed between germlineless animals and animals with an intact reproductive system [17]. However, the transcript levels of the same target genes were reported unaltered upon germ cell removal in a previous study [6]. This dichotomy may be due to different normalization approaches taken by the two studies. Whereas both studies normalized transcript levels to a major housekeeping gene, the first study also took into account the differential number of cells composing animals with an intact gonad and those that lack a germline, whereas the second study did not. Our finding of increased expression of the ATF-7 target gene T24B8.5, obtained by a transcriptional reporter (and thus insensitive to the presence/absence of the germline in terms of normalization) yielded a result consistent with the analysis of Steinbaugh et. al. [17], and suggests that the PMK-1/ATF-7 pathway may also be activated in germlineless animals and may contribute to their immunity. Nevertheless, this pathway may be less critical for the survival of germlineless animals, which can enhance their immune response via alternative transcription factors [6].

In addition to the innate immunity genes *pmk-1* and *atf-7*, the lifespan extension of *irg-7* gof mutants fed with live OP50 depends on the presence of the somatic gonad as well as on a variety of genes required for longevity induced by germ cell removal. This implicates the same systemic endocrine pathways that increase *C*. *elegans* lifespan upon germ cell removal in promoting the resistance of animals to pathogenic challenges. This conclusion is consistent with the expression/activation of many of the genes implicated in the reproductive longevity pathway specifically in the intestine, the same site where the host usually encounters the pathogenic bacteria. Furthermore, in addition to their contribution to germ cell-regulated longevity, both the somatic gonad and the transcription factor DAF-16 have been implicated in the enhanced resistance of germlineless animals to pathogens [6, 69]. Our findings now implicate additional germline-regulated genes such as *tcer-1*, *kri-1*, *daf-12*, *skn-1 and pha-4* in this innate immune response pathway. Interestingly, a bile acid biosynthetic pathway has already been implicated in the systemic communication of cellular stress and activation of the MAP kinase innate immunity pathway by stressed germline [70].

Interestingly, one of the genes required for the longevity of germlineless animals did not affect the longevity of *irg-7 gof* mutants (**Fig 7A**). This gene encodes *nhr-80*, an intestinal transcription factor that transcribes lipid homeostasis-related genes [15]. This differential requirement of *nhr-80* is intriguing and may suggest that *nhr-80* is specifically required for the well-being of germlineless animals rather than an integral part of the reproductive longevity pathway. Nevertheless, this differential requirement of *nhr-80* is important as it genetically rules out the possibility that the *irg-7* gene acts upstream to the germ cells themselves, promoting lifespan extension by limiting the amount of germ cells in the animals. This conclusion is also supported by the fact that in *irg-7 gof* mutants, germline homeostasis and progeny profiles are similar between wild-type animals and *irg-7 gof* mutants (**S1 Fig and Fig 9**).

**Fig 10:**
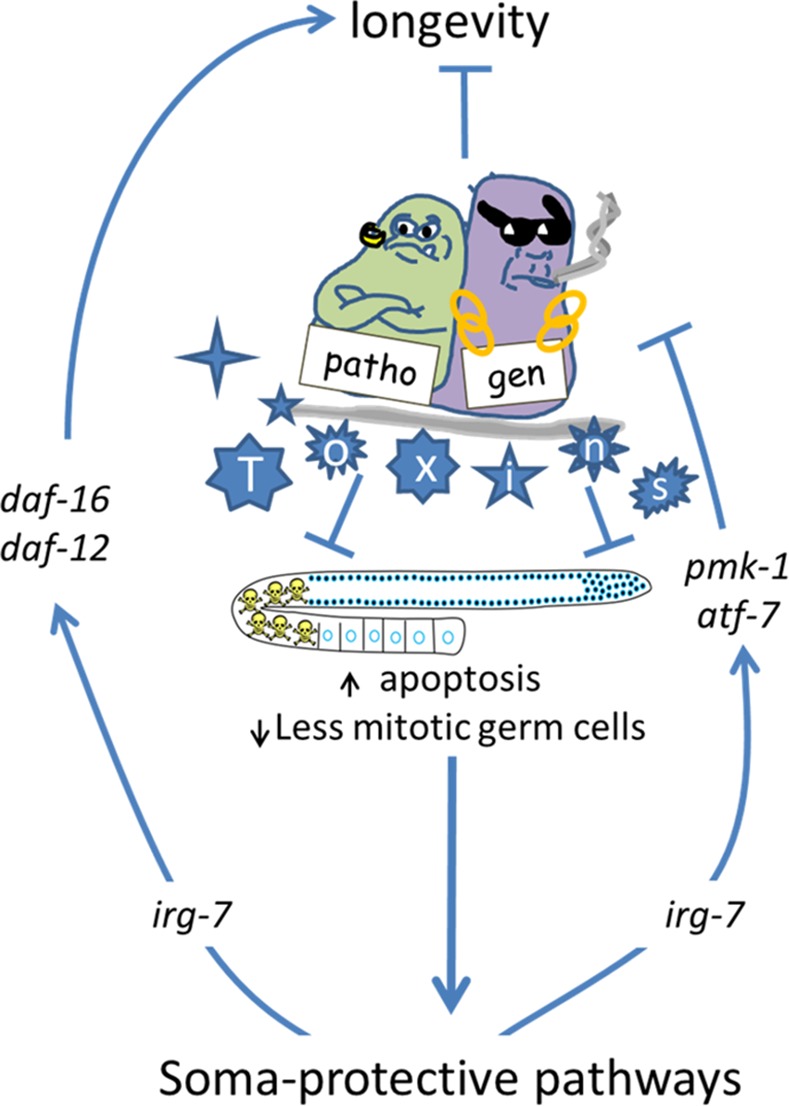
The reproductive innate immunity pathway model. Pathogens are one of the factors that limit lifespan in *C*. *elegans* and in higher organisms. Our data implies that exposure to pathogens and/or their toxins can directly or indirectly modulate germline proliferation and survival resulting in a reproductive system with less germ cells. Germline depletion induces the transcription of *irg-7*, an innate immunity-related secreted protein whose induction promotes the activation of a somatic defense response. On one hand, *irg-7* enables the activation of bona-fida longevity-associated transcription factors, which support the induction of a longevity-promoting transcriptome. At the same time, *irg-7* enables the activation of *atf-7*, a dedicated innate immunity-related transcription factor. In this way, exposure to pathogens can be sensed by perturbations in germline homeostasis. Consequently, the reproductive system can serve as a signaling center to divert key metabolic resources towards defending against the putative pathogen attack by activating the innate immune response.

With the exception of *nhr-80*, the implication of a significant amount of components of the reproductive longevity pathway in pathogen resistance and innate immunity is thought provoking, as it raises the possibility that the reproductive system can be used as a signaling center to divert resources towards defending against putative pathogen attacks (See model in **Fig 10**). Accordingly, perturbations to germ cell homeostasis (executed via regulation of germline proliferation and/or germline apoptosis) may serve as a surveillance center, putatively disrupted by pathogens and their toxins. Such a mode of action is consistent with recent studies that indicate that pathogen surveillance can be achieved indirectly by monitoring internal physiological cues that may be altered by pathogens and their toxins [71–74]; effectively diluting the germline. Accordingly, stress-induced perturbation in germ cell homeostasis can trigger a somatic defense response, including an innate immune response [70, 75].

Consistent with this model, exposure to some pathogens (as previously demonstrated in the case of *Salmonella typhimurium* infection [61] and as shown here for HB luminescence infection) perturbs germ cell homeostasis. In turn, germ cell depletion can activate innate immunity pathways via the expression of the innate immunity-promoting gene *irg-7* **(Fig 6A)**, which in turn can activate the PMK-1/ATF-7 innate immunity pathway **(Fig 4A)**. Likewise, germ cell depletion can promote innate immunity pathways by other means (i.e activation of the transcription factors DAF-16 and SKN-1 in the animals intestine, which in turn induce the expression of a variety of innate immunity-related genes [6, 17].

Finally, in further support of this model, the ability to relay a signal of distress via perturbations in germline homeostasis appears to be important for mounting an effective immune response, as animals are less likely to survive some infections when germline apoptosis is blocked [61 and Fig 9].

One of the hallmarks of aging both in nematodes and in humans, is a progressively increased sensitivity to external pathogens, reflecting a failure of the immune response in the old. At least in *C*. *elegans*, the decline in the ability to combat pathogens is detrimental to the animals and limits their lifespan [76, 77]. Accordingly, it is not surprising that the same manipulations in the reproductive system that promote longevity also affect the animals' innate immune response and increase their resistance to pathogens [6, 29, 30]. However, it is unlikely that the lifespan extension induced by germ cell removal is only the reflection of improved innate immunity. This is because germ cell removal provides additional benefits for the soma and the organism in addition to improving the animals' resistance to pathogens. For example, germ cell depletion maintains proteasome activity [9], prolongs the responsiveness of multiple stress response pathways with age [8, 35] and confers resistance to multiple cellular stresses [7, 78]. Accordingly, the increase in lifespan achieved by the *irg-7 gof* mutation and by feeding the animals with dead bacteria are not as big as that produced by germ-cell loss. Furthermore, *irg-7 gof* only partially phenocopies the transcriptional reprogramming that normally occurs upon germ cell removal. Thus, improved innate immunity is only one of several benefits of animals whose germ cells are depleted. Nevertheless, finding new ways to maintain the immune response in aging animals that undergo immunosensesnce is an important goal that may postpone major lifespan limiting events. Although the immune response in mammals is primarily adaptive in its nature, a basal innate immune response, based on a variety of antiseptic proteins including CTLD proteins, similar to those of the nematode, contributes to the immunity of mammals as well. Thus, better understanding of the molecular events that mediate this basic immune response, in the young and in the old, is important and holds great promise for human health.

## Materials and methods

### Progeny profile

L4 stage N2 or *irg-7(zc6)* worms were incubated at 20°C and transferred to fresh plates twice a day until they stopped producing progeny. Worms that crawled off the plates, bagged or ruptured were removed from the data set. All progeny plates were incubated at 20°C for 2 additional days and the number of worms that developed was determined.

### Plasmids and transgenic animals

The *irg-7* promoter sequence (690 bp) was amplified from genomic DNA and cloned into the HindIII and XbaI sites, replacing a *myo-3* promoter in the pCF191plasmid (previously described in [79]). Germline transformations were performed by injection of 50ng/μl plasmid with 15 ng/μl of *Punc-54*::*gfp* as a co-transformation marker.

### Fluorescence microscopy and quantification

To follow expression of fluorescent proteins, transgenic animals were anaesthetized on 2% agarose pads containing 2mM levamisol. Images were taken with a CCD digital camera using a Nikon 90i fluorescence microscope. For each trial, exposure time was calibrated to minimize the number of saturated pixels and was kept constant throughout the experiment. Nikon NIS element software was used to quantify mean fluorescence intensity in the selected area encompassing whole worms.

### Lifespans

RNAi treatments were performed continuously from the time of hatching unless indicated otherwise. In general, eggs were placed on plates seeded with the RNAi bacteria of interest. Lifespan of 90 animals per strain were scored every 1–2 days. Related lifespans were performed concurrently to minimize variability. In all experiments, lifespan was scored as of the L4 stage which was set as t = 0. Animals that ruptured or crawled off the plates were included in the lifespan analysis as censored worms. For lifespan on dead bacteria, bacteria was killed by 30 minutes of boiling prior to seeding or by UV irradiation. The statistical program SPSS was used to determine the means and the P values calculated using the log-rank (Mantel-Cox) method.

### DAPI staining of dissected gonads

For quantifying the amounts of mitotic germ cells, gonads of day-1 adults were dissected, fixed and stained with DAPI as previously described [80]. In short, day-1 worms were transferred to unseeded (without bacteria) plates and then placed in 20 μl PBSx1 with 0.2mM Levamisole for immobilization. Once the worms were paralyzed, animals were decapitated with a needle to allow the removal of the gonads. The dissected gonads were fixed with 10% formaldehyde for 30 minutes. The fixed gonads were then washed twice in M9 and stained with 1 μg/mL DAPI **(**4',6-diamidino-2-phenylindole) solution for 20 minutes. Worms were washed two times with PBSTx1 and observed under the fluorescent microscope. The boundary of the region of mitotic cells was defined by the most distal row of cells containing nuclei with crescent-shaped DAPI morphology. The amount of mitotic nuclei was scored in sequential focal planes through the width of the germline.

### SYTO12 staining

The number of apoptotic cells in the gonads of day-2 animals was assessed by scoring the number of SYTO12 labeled cells in the gonad. SYTO12 (Molecular Probes) staining was performed as previously described [81]. In short, to obtain an estimate of the relative numbers of corpses in different genetic backgrounds, 2-day adult animals were stained with SYTO 12 (Molecular Probes, Eugene, OR), a vital dye that preferentially stains apoptotic germ cells. Animals were stained by incubating them in a 33 μM aqueous solution of SYTO 12 supplemented with OP50 for 4–5 hours at 25°C. Animals were transferred back to new seeded plates to allow stained bacteria to be purged from the gut. After 30–60 minutes, animals were mounted on agarose pads and inspected using a Nikon eclipse 90i, equipped with standard epifluorescence filters and Nomarski optics. Only animals that stained brightly were scored.

### Pathogen survival

*E*. *faecalis* OG1RF strain (ATCC 47077) was grown at 37 degrees in brain heart infusion (BHI) medium supplemented with Gentamicin (25–50μg/ml). *Photorhabdus luminescens subsp*. *L* strain (ATCC 29999) was grown at 30 degrees for 48 hours in Nutrient Broth medium (BD cat 234000). *Pseudomonas aeruginosa* was the clinical isolate PA14 strain. Bacteria were seeded on nematode growth medium (NGM) plates seeded with bacteria and incubated overnight at room temperature, with the exception of the fast killing experiment by *E*. *faecalis* where the bacteria was seeded on brain heart infusion (BHI) agar plates. Survival assays were initiated with 80-90nematodes in the late L4/early day 1 stage, grown from eggs on plates seeded with OP50 bacteria or RNAi bacteria of interest. Survival was scored daily thereafter. The SPSS program was used to determine the means and the P values. P values were calculated using the log-rank (Mantel-Cox) method.

### Statistical analysis

Error bars represent the standard error of the mean (SEM) of independent biological replicates unless indicated otherwise.

For a simple comparison between two data sets, P values were determined using unpaired **Student’s T-test**, assuming unequal variances.

For multiple comparisons, between multiple data sets, samples were analyzed by the Kruskal-Wallis method, followed by a post hoc analysis using the **Mann-Whitney** method with Bonferroni correction for multiple comparisons. The analysis was performed in R.

To compare pairs of lifespan and pathogen survival analysis (i.e a total of two genotypes), the Kaplan-Meier method was used to estimate survival as a function of time, and survival differences were analyzed by the **Mantel-Cox** log-rank test using the SPSS program.

To compare survival differences between two pairs of genotypes (i.e. a total set of four genotypes), **Cox regression** analysis of several genotype factors was performed to compare hazard ratios between genotypes. Contrasts were used to determine whether the (log) hazard ratio was significantly different under different genotypes. All performed regressions were corrected using the Bonferroni correction for multiple tests. The analysis was performed in R. This analysis is limited to samples with a constant proportional hazard ratio over time.

### Strains, transgenic lines and fosmids

Information provided in S8 Table.

## Supporting information

S1 Fig *irg-7(zc6)* mutants have an overall normal physiology. (A) Average progeny numbers of wild-type animals and *irg-7(zc6)* mutants were assessed twice a day from early adulthood. No significant difference in the progeny profile was observed (P = 0.11). Plot averages 5 independent experiments. (B) Development assay monitoring the developmental rate of wild-type animals (black bars) and *irg-7(zc6)* mutants (grey bars) on OP50 at 20 degrees. Eggs from each genotype were placed on plates. After 48 hours, and every 2 hours intervals thereafter, worms that have reached or passed the L4 stage were scored. Error bars reflect SE of 3 experiments. No less than 90 animals were scored per strain. On average, wild-type animals reached the L4 stage after 48.9 hours (SD = 1.6) whereas *irg-7(zc6)* mutants reached the L4 stage on average after 53.0 hours (SD = 2.5). (C) Average pumping rate of wild-type animals, *irg-7(zc6)* and *eat-2* mutants (known to have a reduced pumping rate) assessed on day 2 of adulthood. No significant difference occurs in the pumping rate between wild-type animals and *irg-7* mutants (Student's t-test values of P = 0.22). 20 animals were analyzed per genotype. Similar results were observed in an additional independent experiment.
(TIF)

S2 Fig Sequences of the *irg-7(zc6)* mutation. Genomic DNA and cDNA from wild-type animals and *irg-7(zc6)* mutants was used as a template for a PCR reaction that encompasses the putative deleted area. The PCR products were then sequenced. Nucleotides present in the wild-type sequence, but not in the mutated sequence are marked in red. Likewise, an insertion of two nucleotides present in the mutant sequence, but not in the wild-type sequence is marked in red. Nucleotides flanking the deletion site are marked in yellow.
(DOCX)

S1 Table Lifespan analysis of *irg-7(zc6)* mutants upon inactivation of ER-stress associated genes. (XLSX)

S2 Table Lifespan effects of f40f4.6 inactivation or overexpression on lifespan. (XLSX)

S3 Table *irg-7* contributes to lifespan extension by improving pathogen resistance. (XLSX)

S4 Table *irg-7* improves pathogen resistance via the PMK-1/ATF-7 innate immunity pathway. (XLSX)

S5 Table Effect of *irg-7* inactivation using *f40f4*.*6* RNAi on the lifespan of longevity mutants. (XLSX)

S6 Table Requirement of known longevity genes and reproductive tissues for lifespan extension by the *irg-7(zc6)* mutation. (XLSX)

S7 Table Germline apoptosis promotes pathogen resistance. (XLSX)

S8 Table Strains, transgenic lines and fosmids. (DOCX)

## References

[1] Hamilton B, Dong Y, Shindo M, Liu W, Odell I, Ruvkun G, et al A systematic RNAi screen for longevity genes in C. elegans. Genes Dev. 2005;19(13):1544–55. 10.1101/gad.1308205 15998808 PMC1172061

[2] Hansen M, Hsu AL, Dillin A, Kenyon C. New genes tied to endocrine, metabolic, and dietary regulation of lifespan from a Caenorhabditis elegans genomic RNAi screen. PLoS Genet. 2005;1(1):119–28. 10.1371/journal.pgen.0010017 16103914 PMC1183531

[3] Kenyon C. The genetics of aging. Nature. 2010;464(7288):504–12. Epub 2010/03/26. 10.1038/nature08980 20336132

[4] Flatt T, Min KJ, D'Alterio C, Villa-Cuesta E, Cumbers J, Lehmann R, et al Drosophila germ-line modulation of insulin signaling and lifespan. Proc Natl Acad Sci U S A. 2008;105(17):6368–73. Epub 2008/04/25. PubMed Central PMCID: PMC2359818. 10.1073/pnas.0709128105 18434551 PMC2359818

[5] Hsin H, Kenyon C. Signals from the reproductive system regulate the lifespan of C. elegans. Nature. 1999;399(6734):362–6. 10.1038/20694 10360574

[6] Alper S, McElwee MK, Apfeld J, Lackford B, Freedman JH, Schwartz DA. The Caenorhabditis elegans germ line regulates distinct signaling pathways to control lifespan and innate immunity. J Biol Chem. 2010;285(3):1822–8. Epub 2009/11/20. PubMed Central PMCID: PMC2804340. 10.1074/jbc.M109.057323 19923212 PMC2804340

[7] Libina N, Berman JR, Kenyon C. Tissue-specific activities of C. elegans DAF-16 in the regulation of lifespan. Cell. 2003;115(4):489–502. 14622602 10.1016/s0092-8674(03)00889-4

[8] Shemesh N, Shai N, Ben-Zvi A. Germline stem cell arrest inhibits the collapse of somatic proteostasis early in Caenorhabditis elegans adulthood. Aging Cell. 2013;12(5):814–22. Epub 2013/06/06. 10.1111/acel.12110 23734734

[9] Vilchez D, Morantte I, Liu Z, Douglas PM, Merkwirth C, Rodrigues AP, et al RPN-6 determines C. elegans longevity under proteotoxic stress conditions. Nature. 2012;489(7415):263–8. Epub 2012/08/28. 10.1038/nature11315 22922647

[10] Lapierre LR, Gelino S, Melendez A, Hansen M. Autophagy and lipid metabolism coordinately modulate life span in germline-less C. elegans. Curr Biol. 2011;21(18):1507–14. Epub 2011/09/13. PubMed Central PMCID: PMC3191188. 10.1016/j.cub.2011.07.042 21906946 PMC3191188

[11] Lapierre LR, Melendez A, Hansen M. Autophagy links lipid metabolism to longevity in C. elegans. Autophagy. 2012;8(1):144–6. Epub 2011/12/22. PubMed Central PMCID: PMC3335999. 10.4161/auto.8.1.18722 22186228 PMC3335999

[12] Wang MC, O'Rourke EJ, Ruvkun G. Fat metabolism links germline stem cells and longevity in C. elegans. Science. 2008;322(5903):957–60. Epub 2008/11/08. PubMed Central PMCID: PMC2760269. 10.1126/science.1162011 18988854 PMC2760269

[13] Ghazi A, Henis-Korenblit S, Kenyon C. Regulation of Caenorhabditis elegans lifespan by a proteasomal E3 ligase complex. Proc Natl Acad Sci U S A. 2007;104(14):5947–52. 10.1073/pnas.0700638104 17392428 PMC1851597

[14] Shen Y, Wollam J, Magner D, Karalay O, Antebi A. A steroid receptor-microRNA switch regulates life span in response to signals from the gonad. Science. 2012;338(6113):1472–6. Epub 2012/12/15. PubMed Central PMCID: PMC3909774. 10.1126/science.1228967 23239738 PMC3909774

[15] Goudeau J, Bellemin S, Toselli-Mollereau E, Shamalnasab M, Chen Y, Aguilaniu H. Fatty acid desaturation links germ cell loss to longevity through NHR-80/HNF4 in C. elegans. PLoS Biol. 2011;9(3):e1000599 Epub 2011/03/23. PubMed Central PMCID: PMC3057950. 10.1371/journal.pbio.1000599 21423649 PMC3057950

[16] O'Rourke EJ, Ruvkun G. MXL-3 and HLH-30 transcriptionally link lipolysis and autophagy to nutrient availability. Nat Cell Biol. 2013;15(6):668–76. Epub 2013/04/23. PubMed Central PMCID: PMC3723461. 10.1038/ncb2741 23604316 PMC3723461

[17] Steinbaugh MJ, Narasimhan SD, Robida-Stubbs S, Moronetti Mazzeo LE, Dreyfuss JM, Hourihan JM, et al Lipid-mediated regulation of SKN-1/Nrf in response to germ cell absence. eLife. 2015;4. Epub 2015/07/22. PubMed Central PMCID: PMC4541496. 10.7554/eLife.07836 PMC4541496 26196144

[18] Ratnappan R, Amrit FR, Chen SW, Gill H, Holden K, Ward J, et al Germline signals deploy NHR-49 to modulate fatty-acid β-oxidation and desaturation in somatic tissues of C. elegans. PLoS Genet. 2014;10(12):e1004829 PubMed Central PMCID: PMCPMC4256272. 10.1371/journal.pgen.1004829 25474470 PMC4256272

[19] Kenyon C. A pathway that links reproductive status to lifespan in Caenorhabditis elegans. Ann N Y Acad Sci. 2010;1204:156–62. Epub 2010/08/27. 10.1111/j.1749-6632.2010.05640.x 20738286

[20] Shivers RP, Pagano DJ, Kooistra T, Richardson CE, Reddy KC, Whitney JK, et al Phosphorylation of the conserved transcription factor ATF-7 by PMK-1 p38 MAPK regulates innate immunity in Caenorhabditis elegans. PLoS Genet. 2010;6(4):e1000892 Epub 2010/04/07. PubMed Central PMCID: PMC2848548. 10.1371/journal.pgen.1000892 20369020 PMC2848548

[21] Kim DH, Feinbaum R, Alloing G, Emerson FE, Garsin DA, Inoue H, et al A conserved p38 MAP kinase pathway in Caenorhabditis elegans innate immunity. Science. 2002;297(5581):623–6. Epub 2002/07/27. 10.1126/science.1073759 12142542

[22] Garigan D, Hsu AL, Fraser AG, Kamath RS, Ahringer J, Kenyon C. Genetic analysis of tissue aging in Caenorhabditis elegans: a role for heat-shock factor and bacterial proliferation. Genetics. 2002;161(3):1101–12. 12136014 10.1093/genetics/161.3.1101 PMC1462187

[23] Herndon LA, Schmeissner PJ, Dudaronek JM, Brown PA, Listner KM, Sakano Y, et al Stochastic and genetic factors influence tissue-specific decline in ageing C. elegans. Nature. 2002;419(6909):808–14. 10.1038/nature01135 12397350

[24] McGee MD, Weber D, Day N, Vitelli C, Crippen D, Herndon LA, et al Loss of intestinal nuclei and intestinal integrity in aging C. elegans. Aging Cell. 2011;10(4):699–710. Epub 2011/04/20. PubMed Central PMCID: PMC3135675. 10.1111/j.1474-9726.2011.00713.x 21501374 PMC3135675

[25] Pujol N, Cypowyj S, Ziegler K, Millet A, Astrain A, Goncharov A, et al Distinct innate immune responses to infection and wounding in the C. elegans epidermis. Curr Biol. 2008;18(7):481–9. Epub 2008/04/09. PubMed Central PMCID: PMC2394561. 10.1016/j.cub.2008.02.079 18394898 PMC2394561

[26] Gems D, Riddle DL. Genetic, behavioral and environmental determinants of male longevity in Caenorhabditis elegans. Genetics. 2000;154(4):1597–610. Epub 2000/04/04. PubMed Central PMCID: PMC1461011. 10747056 10.1093/genetics/154.4.1597 PMC1461011

[27] Miyata S, Begun J, Troemel ER, Ausubel FM. DAF-16-dependent suppression of immunity during reproduction in Caenorhabditis elegans. Genetics. 2008;178(2):903–18. Epub 2008/02/05. PubMed Central PMCID: PMC2248360. 10.1534/genetics.107.083923 18245330 PMC2248360

[28] Evans EA, Chen WC, Tan MW. The DAF-2 insulin-like signaling pathway independently regulates aging and immunity in C. elegans. Aging Cell. 2008;7(6):879–93. Epub 2008/09/11. PubMed Central PMCID: PMC2630471. 10.1111/j.1474-9726.2008.00435.x 18782349 PMC2630471

[29] Garsin DA, Villanueva JM, Begun J, Kim DH, Sifri CD, Calderwood SB, et al Long-lived C. elegans daf-2 mutants are resistant to bacterial pathogens. Science. 2003;300(5627):1921 Epub 2003/06/21. 10.1126/science.1080147 12817143

[30] Pellegrino MW, Nargund AM, Kirienko NV, Gillis R, Fiorese CJ, Haynes CM. Mitochondrial UPR-regulated innate immunity provides resistance to pathogen infection. Nature. 2014;516(7531):414–7. Epub 2014/10/03. PubMed Central PMCID: PMC4270954. 10.1038/nature13818 25274306 PMC4270954

[31] McElwee J, Bubb K, Thomas JH. Transcriptional outputs of the Caenorhabditis elegans forkhead protein DAF-16. Aging Cell. 2003;2(2):111–21. 12882324 10.1046/j.1474-9728.2003.00043.x

[32] Murphy CT, McCarroll SA, Bargmann CI, Fraser A, Kamath RS, Ahringer J, et al Genes that act downstream of DAF-16 to influence the lifespan of Caenorhabditis elegans. Nature. 2003;424(6946):277–83. 10.1038/nature01789 12845331

[33] Troemel ER, Chu SW, Reinke V, Lee SS, Ausubel FM, Kim DH. p38 MAPK regulates expression of immune response genes and contributes to longevity in C. elegans. PLoS Genet. 2006;2(11):e183 Epub 2006/11/14. PubMed Central PMCID: PMC1635533. 10.1371/journal.pgen.0020183 17096597 PMC1635533

[34] Ben-Zvi A, Miller EA, Morimoto RI. Collapse of proteostasis represents an early molecular event in Caenorhabditis elegans aging. Proc Natl Acad Sci U S A. 2009;106(35):14914–9. 10.1073/pnas.0902882106 19706382 PMC2736453

[35] Labbadia J, Morimoto RI. Repression of the Heat Shock Response Is a Programmed Event at the Onset of Reproduction. Mol Cell. 2015;59(4):639–50. Epub 2015/07/28. PubMed Central PMCID: PMC4546525. 10.1016/j.molcel.2015.06.027 26212459 PMC4546525

[36] Taylor RC, Dillin A. XBP-1 is a cell-nonautonomous regulator of stress resistance and longevity. Cell. 2013;153(7):1435–47. Epub 2013/06/26. 10.1016/j.cell.2013.05.042 23791175 PMC4771415

[37] Calfon M, Zeng H, Urano F, Till JH, Hubbard SR, Harding HP, et al IRE1 couples endoplasmic reticulum load to secretory capacity by processing the XBP-1 mRNA. Nature. 2002;415(6867):92–6. 10.1038/415092a 11780124

[38] Doitsidou M, Poole RJ, Sarin S, Bigelow H, Hobert O. C. elegans mutant identification with a one-step whole-genome-sequencing and SNP mapping strategy. PLoS One. 2010;5(11):e15435 Epub 2010/11/17. PubMed Central PMCID: PMC2975709. 10.1371/journal.pone.0015435 21079745 PMC2975709

[39] Engelmann I, Griffon A, Tichit L, Montañana-Sanchis F, Wang G, Reinke V, et al A comprehensive analysis of gene expression changes provoked by bacterial and fungal infection in C. elegans. PLoS One. 2011;6(5):e19055 PubMed Central PMCID: PMCPMC3094335. 10.1371/journal.pone.0019055 21602919 PMC3094335

[40] Ludewig AH, Klapper M, Döring F. Identifying evolutionarily conserved genes in the dietary restriction response using bioinformatics and subsequent testing in Caenorhabditis elegans. Genes Nutr. 2014;9(1):363 PubMed Central PMCID: PMCPMC3896620. 10.1007/s12263-013-0363-5 24311442 PMC3896620

[41] Schulenburg H, Hoeppner MP, Weiner J 3rd, Bornberg-Bauer E. Specificity of the innate immune system and diversity of C-type lectin domain (CTLD) proteins in the nematode Caenorhabditis elegans. Immunobiology. 2008;213(3–4):237–50. Epub 2008/04/15. 10.1016/j.imbio.2007.12.004 18406370

[42] Mukherjee S, Zheng H, Derebe MG, Callenberg KM, Partch CL, Rollins D, et al Antibacterial membrane attack by a pore-forming intestinal C-type lectin. Nature. 2014;505(7481):103–7. Epub 2013/11/22. PubMed Central PMCID: PMC4160023. 10.1038/nature12729 24256734 PMC4160023

[43] Vaishnava S, Yamamoto M, Severson KM, Ruhn KA, Yu X, Koren O, et al The antibacterial lectin RegIIIgamma promotes the spatial segregation of microbiota and host in the intestine. Science. 2011;334(6053):255–8. Epub 2011/10/15. PubMed Central PMCID: PMC3321924. 10.1126/science.1209791 21998396 PMC3321924

[44] Richardson CE, Kooistra T, Kim DH. An essential role for XBP-1 in host protection against immune activation in C. elegans. Nature. 2010;463(7284):1092–5. Epub 2010/02/26. PubMed Central PMCID: PMC2834299. 10.1038/nature08762 20182512 PMC2834299

[45] Lin K, Hsin H, Libina N, Kenyon C. Regulation of the Caenorhabditis elegans longevity protein DAF-16 by insulin/IGF-1 and germline signaling. Nat Genet. 2001;28(2):139–45. 10.1038/88850 11381260

[46] McCormick M, Chen K, Ramaswamy P, Kenyon C. New genes that extend Caenorhabditis elegans' lifespan in response to reproductive signals. Aging Cell. 2012;11(2):192–202. Epub 2011/11/16. PubMed Central PMCID: PMC4342234. 10.1111/j.1474-9726.2011.00768.x 22081913 PMC4342234

[47] Yamawaki TM, Berman JR, Suchanek-Kavipurapu M, McCormick M, Gaglia MM, Lee SJ, et al The somatic reproductive tissues of C. elegans promote longevity through steroid hormone signaling. PLoS Biol. 2010;8(8). Epub 2010/09/09. PubMed Central PMCID: PMC2930862. 10.1371/journal.pbio.1000468 PMC2930862 20824162

[48] Yen K, Narasimhan SD, Tissenbaum HA. DAF-16/Forkhead box O transcription factor: many paths to a single Fork(head) in the road. Antioxid Redox Signal. 2011;14(4):623–34. PubMed Central PMCID: PMCPMC3021330. 10.1089/ars.2010.3490 20673162 PMC3021330

[49] Ghazi A, Henis-Korenblit S, Kenyon C. A transcription elongation factor that links signals from the reproductive system to lifespan extension in Caenorhabditis elegans. PLoS Genet. 2009;5(9):e1000639 10.1371/journal.pgen.1000639 19749979 PMC2729384

[50] Berman JR, Kenyon C. Germ-cell loss extends C. elegans life span through regulation of DAF-16 by kri-1 and lipophilic-hormone signaling. Cell. 2006;124(5):1055–68. 10.1016/j.cell.2006.01.039 16530050

[51] Bishop NA, Guarente L. Two neurons mediate diet-restriction-induced longevity in C. elegans. Nature. 2007;447(7144):545–9. 10.1038/nature05904 17538612

[52] Tullet JM, Hertweck M, An JH, Baker J, Hwang JY, Liu S, et al Direct inhibition of the longevity-promoting factor SKN-1 by insulin-like signaling in C. elegans. Cell. 2008;132(6):1025–38. 10.1016/j.cell.2008.01.030 18358814 PMC2367249

[53] Ogg S, Paradis S, Gottlieb S, Patterson GI, Lee L, Tissenbaum HA, et al The Fork head transcription factor DAF-16 transduces insulin-like metabolic and longevity signals in C. elegans. Nature. 1997;389(6654):994–9. 10.1038/40194 9353126

[54] Kenyon C, Chang J, Gensch E, Rudner A, Tabtiang R. A C. elegans mutant that lives twice as long as wild type. Nature. 1993;366(6454):461–4. 10.1038/366461a0 8247153

[55] Hsu AL, Murphy CT, Kenyon C. Regulation of aging and age-related disease by DAF-16 and heat-shock factor. Science. 2003;300(5622):1142–5. 10.1126/science.1083701 12750521

[56] Apfeld J, O'Connor G, McDonagh T, DiStefano PS, Curtis R. The AMP-activated protein kinase AAK-2 links energy levels and insulin-like signals to lifespan in C. elegans. Genes Dev. 2004;18(24):3004–9. 10.1101/gad.1255404 15574588 PMC535911

[57] Panowski SH, Wolff S, Aguilaniu H, Durieux J, Dillin A. PHA-4/Foxa mediates diet-restriction-induced longevity of C. elegans. Nature. 2007;447(7144):550–5. 10.1038/nature05837 17476212

[58] Durieux J, Wolff S, Dillin A. The cell-non-autonomous nature of electron transport chain-mediated longevity. Cell. 2011;144(1):79–91. Epub 2011/01/11. PubMed Central PMCID: PMC3062502. 10.1016/j.cell.2010.12.016 21215371 PMC3062502

[59] Cristina D, Cary M, Lunceford A, Clarke C, Kenyon C. A regulated response to impaired respiration slows behavioral rates and increases lifespan in Caenorhabditis elegans. PLoS Genet. 2009;5(4):e1000450 10.1371/journal.pgen.1000450 19360127 PMC2660839

[60] Carrano AC, Liu Z, Dillin A, Hunter T. A conserved ubiquitination pathway determines longevity in response to diet restriction. Nature. 2009;460(7253):396–9. Epub 2009/06/26. PubMed Central PMCID: PMC2746748. 10.1038/nature08130 19553937 PMC2746748

[61] Aballay A, Ausubel FM. Programmed cell death mediated by ced-3 and ced-4 protects Caenorhabditis elegans from Salmonella typhimurium-mediated killing. Proc Natl Acad Sci U S A. 2001;98(5):2735–9. Epub 2001/02/28. PubMed Central PMCID: PMC30208. 10.1073/pnas.041613098 11226309 PMC30208

[62] Ghosh D, Porter E, Shen B, Lee SK, Wilk D, Drazba J, et al Paneth cell trypsin is the processing enzyme for human defensin-5. Nat Immunol. 2002;3(6):583–90. Epub 2002/05/22. 10.1038/ni797 12021776

[63] Wilson CL, Ouellette AJ, Satchell DP, Ayabe T, Lopez-Boado YS, Stratman JL, et al Regulation of intestinal alpha-defensin activation by the metalloproteinase matrilysin in innate host defense. Science. 1999;286(5437):113–7. Epub 1999/10/03. 10506557 10.1126/science.286.5437.113

[64] Kawli T, Tan MW. Neuroendocrine signals modulate the innate immunity of Caenorhabditis elegans through insulin signaling. Nat Immunol. 2008;9(12):1415–24. Epub 2008/10/16. 10.1038/ni.1672 18854822

[65] Zugasti O, Ewbank JJ. Neuroimmune regulation of antimicrobial peptide expression by a noncanonical TGF-beta signaling pathway in Caenorhabditis elegans epidermis. Nat Immunol. 2009;10(3):249–56. Epub 2009/02/10. 10.1038/ni.1700 19198592

[66] Miltsch SM, Seeberger PH, Lepenies B. The C-type lectin-like domain containing proteins Clec-39 and Clec-49 are crucial for Caenorhabditis elegans immunity against Serratia marcescens infection. Dev Comp Immunol. 2014;45(1):67–73. 10.1016/j.dci.2014.02.002 24534554

[67] O'Rourke D, Baban D, Demidova M, Mott R, Hodgkin J. Genomic clusters, putative pathogen recognition molecules, and antimicrobial genes are induced by infection of C. elegans with M. nematophilum. Genome Res. 2006;16(8):1005–16. PubMed Central PMCID: PMCPMC1524860. 10.1101/gr.50823006 16809667 PMC1524860

[68] Shapira M, Hamlin BJ, Rong J, Chen K, Ronen M, Tan MW. A conserved role for a GATA transcription factor in regulating epithelial innate immune responses. Proc Natl Acad Sci U S A. 2006;103(38):14086–91. PubMed Central PMCID: PMCPMC1599916. 10.1073/pnas.0603424103 16968778 PMC1599916

[69] Rae R, Sinha A, Sommer RJ. Genome-wide analysis of germline signaling genes regulating longevity and innate immunity in the nematode Pristionchus pacificus. PLoS pathogens. 2012;8(8):e1002864 Epub 2012/08/23. PubMed Central PMCID: PMC3415453. 10.1371/journal.ppat.1002864 22912581 PMC3415453

[70] Govindan JA, Jayamani E, Zhang X, Breen P, Larkins-Ford J, Mylonakis E, et al Lipid signalling couples translational surveillance to systemic detoxification in Caenorhabditis elegans. Nat Cell Biol. 2015;17(10):1294–303. Epub 2015/09/01. PubMed Central PMCID: PMC4589496. 10.1038/ncb3229 26322678 PMC4589496

[71] Melo JA, Ruvkun G. Inactivation of conserved C. elegans genes engages pathogen- and xenobiotic-associated defenses. Cell. 2012;149(2):452–66. Epub 2012/04/17. 10.1016/j.cell.2012.02.050 22500807 PMC3613046

[72] Meisel JD, Panda O, Mahanti P, Schroeder FC, Kim DH. Chemosensation of bacterial secondary metabolites modulates neuroendocrine signaling and behavior of C. elegans. Cell. 2014;159(2):267–80. PubMed Central PMCID: PMCPMC4194030. 10.1016/j.cell.2014.09.011 25303524 PMC4194030

[73] Korta DZ, Tuck S, Hubbard EJ. S6K links cell fate, cell cycle and nutrient response in C. elegans germline stem/progenitor cells. Development. 2012;139(5):859–70. Epub 2012/01/27. PubMed Central PMCID: PMC3274352. 10.1242/dev.074047 22278922 PMC3274352

[74] Michaelson D, Korta DZ, Capua Y, Hubbard EJ. Insulin signaling promotes germline proliferation in C. elegans. Development. 2010;137(4):671–80. Epub 2010/01/30. PubMed Central PMCID: PMC2827619. 10.1242/dev.042523 20110332 PMC2827619

[75] Ermolaeva MA, Segref A, Dakhovnik A, Ou HL, Schneider JI, Utermohlen O, et al DNA damage in germ cells induces an innate immune response that triggers systemic stress resistance. Nature. 2013;501(7467):416–20. Epub 2013/08/27. 10.1038/nature12452 23975097 PMC4120807

[76] Laws TR, Harding SV, Smith MP, Atkins TP, Titball RW. Age influences resistance of Caenorhabditis elegans to killing by pathogenic bacteria. FEMS microbiology letters. 2004;234(2):281–7. Epub 2004/05/12. 10.1016/j.femsle.2004.03.034 15135534

[77] Youngman MJ, Rogers ZN, Kim DH. A decline in p38 MAPK signaling underlies immunosenescence in Caenorhabditis elegans. PLoS Genet. 2011;7(5):e1002082 Epub 2011/06/01. PubMed Central PMCID: PMC3098197. 10.1371/journal.pgen.1002082 21625567 PMC3098197

[78] Yamawaki TM, Arantes-Oliveira N, Berman JR, Zhang P, Kenyon C. Distinct activities of the germline and somatic reproductive tissues in the regulation of Caenorhabditis elegans' longevity. Genetics. 2008;178(1):513–26. 10.1534/genetics.107.083253 18202391 PMC2206098

[79] Zhang P, Judy M, Lee SJ, Kenyon C. Direct and indirect gene regulation by a life-extending FOXO protein in C. elegans: roles for GATA factors and lipid gene regulators. Cell Metab. 2013;17(1):85–100. Epub 2013/01/15. PubMed Central PMCID: PMC3969420. 10.1016/j.cmet.2012.12.013 23312285 PMC3969420

[80] Francis R, Maine E, Schedl T. Analysis of the multiple roles of gld-1 in germline development: interactions with the sex determination cascade and the glp-1 signaling pathway. Genetics. 1995;139(2):607–30. Epub 1995/02/01. PubMed Central PMCID: PMC1206369. 7713420 10.1093/genetics/139.2.607 PMC1206369

[81] Gumienny TL, Lambie E, Hartwieg E, Horvitz HR, Hengartner MO. Genetic control of programmed cell death in the Caenorhabditis elegans hermaphrodite germline. Development. 1999;126(5):1011–22. 9927601 10.1242/dev.126.5.1011

