# Supplementary figures and images for: Innate immunity mediated longevity and longevity induced by germ cell removal converge on the C-type lectin domain protein IRG-7
Source: PLoS Genet. 2017 Feb 14;13(2):e1006577. doi: 10.1371/journal.pgen.1006577 (PMC5308781; doi:10.1371/journal.pgen.1006577)

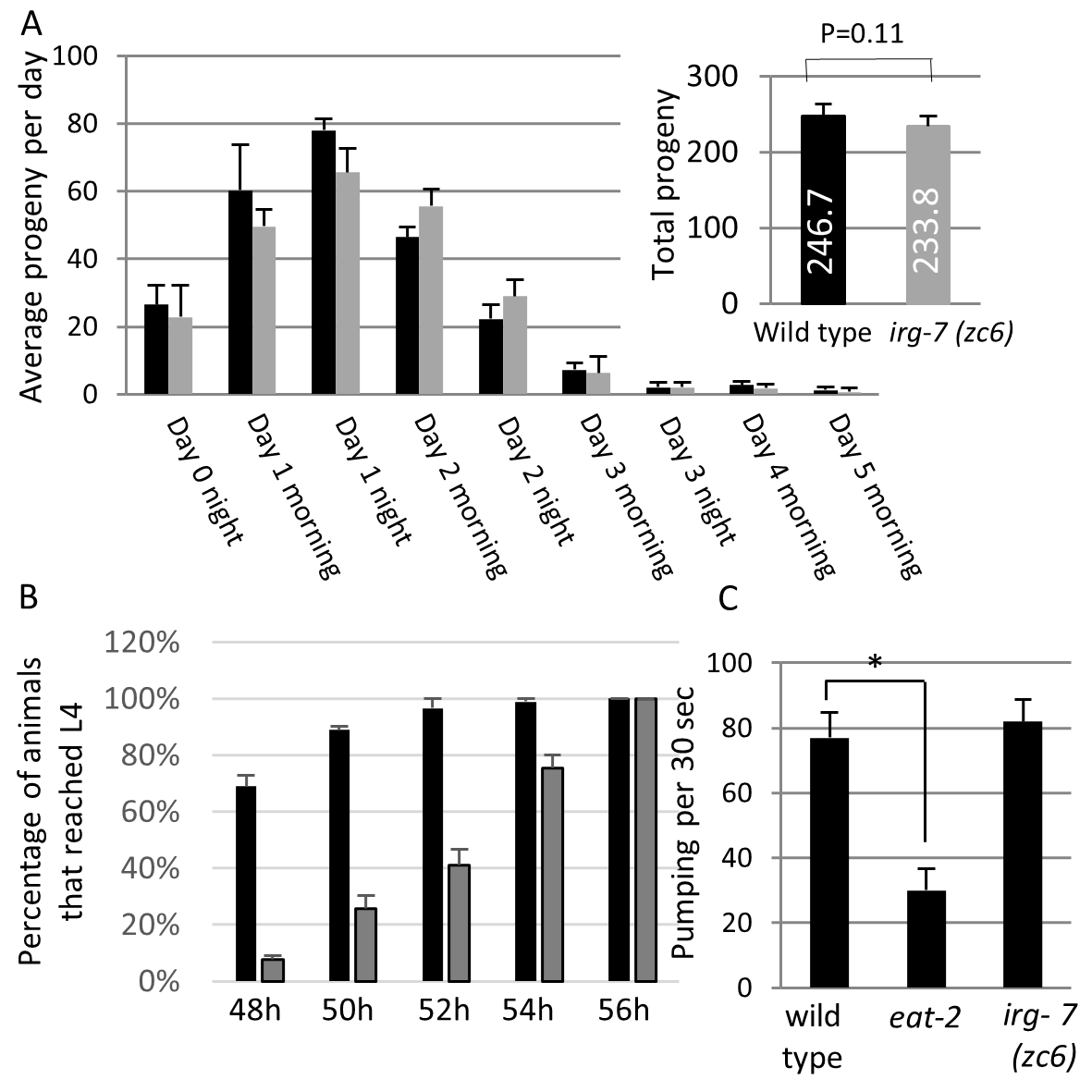

Supplement: S1 Fig — (A) Average progeny numbers of wild-type animals and irg-7(zc6) mutants were assessed twice a day from early adulthood. No significant difference in the progeny profile was observed (P = 0.11). Plot averages 5 independent experiments. (B) Development assay monitoring the developmental rate of wild-type animals (black bars) and irg-7(zc6) mutants (grey bars) on OP50 at 20 degrees. Eggs from each genotype were placed on plates. After 48 hours, and every 2 hours intervals thereafter, worms that have reached or passed the L4 stage were scored. Error bars reflect SE of 3 experiments. No less than 90 animals were scored per strain. On average, wild-type animals reached the L4 stage after 48.9 hours (SD = 1.6) whereas irg-7(zc6) mutants reached the L4 stage on average after 53.0 hours (SD = 2.5). (C) Average pumping rate of wild-type animals, irg-7(zc6) and eat-2 mutants (known to have a reduced pumping rate) assessed on day 2 of adulthood. No significant difference occurs in the pumping rate between wild-type animals and irg-7 mutants (Student's t-test values of P = 0.22). 20 animals were analyzed per genotype. Similar results were observed in an additional independent experiment. (TIF) [file pgen.1006577.s001.tif]
