# Supplementary material for: Innate immunity mediated longevity and longevity induced by germ cell removal converge on the C-type lectin domain protein IRG-7
Source: PLoS Genet. 2017 Feb 14;13(2):e1006577. doi: 10.1371/journal.pgen.1006577 (PMC5308781; doi:10.1371/journal.pgen.1006577)
Supplement: S2 Fig — Genomic DNA and cDNA from wild-type animals and irg-7(zc6) mutants was used as a template for a PCR reaction that encompasses the putative deleted area. The PCR products were then sequenced. Nucleotides present in the wild-type sequence, but not in the mutated sequence are marked in red. Likewise, an insertion of two nucleotides present in the mutant sequence, but not in the wild-type sequence is marked in red. Nucleotides flanking the deletion site are marked in yellow. (DOCX) [file pgen.1006577.s002.docx]

**Genomic wild type *irg-7*, with 250 bp flanks**:

red = mutation 
yellow = flanking sequence

**Genomic *irg-7(zc6),* with 250 bp flanks**:

**cDNA wild type *irg-7*, with flanking sequences:**

 

**cDNA *irg-7(zc6)*, with flankng sequences:**
