# Supplementary material for: Innate immunity mediated longevity and longevity induced by germ cell removal converge on the C-type lectin domain protein IRG-7
Source: PLoS Genet. 2017 Feb 14;13(2):e1006577. doi: 10.1371/journal.pgen.1006577 (PMC5308781; doi:10.1371/journal.pgen.1006577)
Supplement: S8 Table — (DOCX) [file pgen.1006577.s010.docx]

**List of strains used in this study**

| Strain name | **Genotype** | **Notes** |
| --- | --- | --- |
| N2 |  | **Kenyon lab** |
| CB4856 |  | **CGC** |
| SHK82 | *irg-7(zc6)* *X* | **Outcrossed fromSJ6** |
| CF1903 | *glp-1(e2144) III* | **Kenyon lab** |
| CF1041 | *daf-2(e1370*) *III* | **Kenyon lab** |
| CF1908 | *eat-2(ad1116*) *II* | **Kenyon lab** |
| CF2354 | *clk-1(qm30) II* | **Kenyon lab** |
| CF2012 | *pek-1(ok275) X* | **Kenyon lab** |
| CF2988 | *atf-6(ok551)* *X* | **Kenyon lab** |
| CF2479 | *daf-12(rh61rh411) X* | **Kenyon lab** |
| CF1037 | *daf-16(mu86) I* | **Kenyon lab** |
| BX165 | *nhr-80(tm1011) III* | **CGC** |
| CF2253 | *gon-2(q388) I* | **Kenyon lab** |
| VC1518 | *atf-7(gk715) III* | **CGC** |
|  |  |  |
|  | ***Transgenic strains*** |  |
| CF2260 | *zcIs4 [Phsp-4::*GFP*]* *V* | **Kenyon lab, outcrossed from SJ4005** |
| CF2258 | *zcIs4 [Phsp-4::*GFP*] V; irg-7(zc6) X* | **Outcrossed from SJ6** |
| SHK253 | *zcIs4 [Phsp-4::*GFP*]* V*; irg-7(zc6) pek-1(ok275) X* | **CF2258XCF2012** |
| SHK252 | *zcIs4 [Phsp-4::*GFP*] V; irg-7(zc6)* *atf-6(ok551)* *X* | **CF2258XCF2988** |
| SHK90 | *zcIs4 [Phsp-4::*GFP*] V; irg-7(zc6)* *daf-12(rh61rh411) X* | **CF2258XCF2479** |
| SHK324 | *daf-16(mu86) I*; *zcIs4 [Phsp-4::*GFP*] V*; *irg-7(zc6) X* | **CF2258XCF1037** |
| SHK92 | *nhr-80(tm1011) III*; *zcIs4 [Phsp-4::*GFP*]* V; *irg-7(zc6) X* | **CF2258XBX165** |
| SHK-44 | *glp-1(e2144)III ; zcIs4 [Phsp-4::*GFP*] V; irg-7(zc6) X* | **CF2258XCF1903** |
| SHK42 | *gon-2(q388) I; zcIs4 [Phsp-4::*GFP*] V;* *irg-7(zc6) X* | **CF2258XCF2253** |
| SHK275 | *atf-7(gk715) III; zcIs4 [Phsp-4::*GFP*] V; irg-7(zc6) X* | **CF2258XVC1518** |
| CF1553 | muIs84[*Pad76 sod-3*::GFP] | **Kenyon lab** |
| CF1929 | *glp-1(e2144) III*; *muIs84[Pad76 sod-3*::GFP] | **Kenyon lab** |
| SHK121 | *muIs84[Pad76 Psod-3::GFP]; irg-7*. *(zc6) X* | **CF1553XSHK82** |
| BC15369 | *dpy-5(e907) I*; *sEx15369[Pcdr-6*/rCesK01D12.12::GFP + *pCeh361(dpy-5*(+))] | **CGC** |
| SHK81 | *glp-1(e2144)III; sEx15369*[*Pcdr-6/rCesK01D12.12*::GFP + *pCeh361(dpy-5(+*))]*;* | **BC15369XCF1903XCF1037** |
| SHK101 | *dpy-5(e907) I*; *sEx15369*[*Pcdr-6/rCesK01D12.12*::GFP *+ pCeh361(dpy-5*(+))]; *daf-16(mu86); glp-1(e2144) III* | **BC15369XCF1903** |
| SHK154 | *dpy-5(e907)* *I*; *sEx15369[Pcdr-6/rCesK01D12.12::*GFP *+ pCeh361(dpy-5(+))]; irg-7(zc6) X* | **BC15369XSHK82** |
| SHK254 | *dpy-5(e907)* *I*; *sEx15369[Pcdr-6/rCesK01D12.12::*GFP *+ pCeh361(dpy-5(+*))]; *irg-7(zc6) daf-12(rh61rh411) X* | **SHK154XCF2479** |
| SHK255 | *dpy-5(e907) I*; *daf-12(rh61rh411) X*; sEx15369[*Pcdr-6*/rCesK01D12.12::GFP + pCeh361(*dpy-5(+)*] | **BC15369XCF2479** |
| DA2123 | *adIs2122[lgg-1*::GFP + *rol-6*] | **CGC** |
| MAH44 | *glp-1(e2144) III*; *adIs2122[lgg-1*::GFP + *rol-6*] | **CGC** |
| BX115 | *lin-15B(n765*)*X* ; *waEx16 [fat-6::GFP + lin15(+)]* | **CGC** |
| SHK95: | *glp-1(e2144) III; lin-15B(n765*)X ; *waEx16 [fat-6::GFP + lin15(+)]* | **BX115XCF1903** |
| SHK113 | BiuEx8[*P irg-7:*:NLS::RFP+*Punc-54*::GFP] |  |
| SHK122 | *glp-1(e2144) III* ; BiuEx8*[P irg-7::*NLS:*:*RFP*+Punc-54::*GFP*]* | **SHK113XCF1903** |
| SHK123 | *irg-7(zc6)* *X*; BiuEx8*[P irg-7::*NLS::RFP*+Punc-54::*GFP*]* | **SHK113XSHK82** |
| AU78 | *agIs219[T24B8.5p::*GFP*::unc-54-3' UTR + ttx-3p::*GFP*::unc-54-3' UTR] III* | **CGC** |
| SHK273 | *agIs219[T24B8.5p::*GFP*::unc-54-3' UTR + ttx-3p::*GFP*::unc-54-3' UTR] III* ;  *irg-7(zc6) X* | **AU78XSHK82** |
|  |  |  |
| CF1934 | *daf-16(mu86) I*; *muIs109*[GFP::DAF-16cDNA; *Podr1*::RFP] | **Kenyon lab** |
| CF1935 | *daf-16(mu86) I; glp-1(e2144)*; *muIs109*[GFP::DAF-16cDNA; *Podr1*::RFP] | **Kenyon lab** |
| SHK120 | *daf-16(mu86) I;glp-1(e2144)* *III*; *muIs109*[GFP::DAF-16cDNA; *Podr1*::RFP]*; irg-7(zc6)* X | **CF1935XSHK82** |
| SHK292 | *biuEx14[Pgrd-10::gfp*(50 ng/μl) +WRM0619aB08(10ng/μl)] | Fosmid WRM0619aB08 was a kind gift from Oliver Hobert. |
| SHK294 | *biuEx15[Pgrd-10::gfp(*50 ng/μl) +WRM0635bF05(10ng/μl)] | Fosmid WRM0635bF05 was a kind gift from Oliver Hobert. |
